# Key Methodologies in Characterizing the Multi-Scale Structures of Gluten Proteins in Dough: A Comparative Review

**DOI:** 10.3390/biom16030382

**Published:** 2026-03-03

**Authors:** Feifei Su, Yiyuan Zou, Zehua Zhang, Zhiling Tang, Haoran Luo, Fayin Ye, Guohua Zhao

**Affiliations:** 1 College of Food Science, Southwest University, 2 Tiansheng Road, Chongqing 400715, China; sufeifei951@email.swu.edu.cn (F.S.); zyy2021@email.swu.edu.cn (Y.Z.); zhangzehua1995119@gmail.com (Z.Z.); tangzhiling@email.swu.edu.cn (Z.T.); lmice221218@gmail.com (H.L.); fye@swu.edu.cn (F.Y.); 2 Chongqing Key Laboratory of Speciality Food Co-Built by Sichuan and Chongqing, Chongqing 400715, China; 3 College of Life Science, Sichuan Normal University, Chengdu 610101, China

**Keywords:** gluten aggregates, gluten network, dough formation, in-situ detection, structural parameters

## Abstract

Gluten proteins are key components in wheat flour that determine the formation of dough and the quality of flour-based products. Upon hydration and mixing, gluten proteins undergo complex structural transformations to form a gluten network, exhibiting a hierarchical multi-scale structure spanning molecular, aggregate, and network scales. Due to the extreme complexity of gluten proteins, accurately characterizing their multi-scale structures remains challenging, requiring the combined application of multiple techniques, which are still relatively limited and thus warrant further exploration. Therefore, this review presents the principles, operational details, and result presentations of current techniques at different structural scales, including electrophoresis, high-performance liquid chromatography, proteomics, Fourier transform infrared spectroscopy, and Fourier transform Raman spectroscopy at the molecular scale; size-exclusion chromatography, asymmetrical flow field-flow fractionation, dynamic light scattering, multi-angle light scattering, differential refractive index, and ultraviolet absorbance at the aggregate scale; and confocal laser scanning microscopy, scanning electron microscopy, confocal Raman microscopy, and two-photon excitation microscopy at the network scale, among others. It further compares the advantages and disadvantages of similar techniques, facilitating their scenario-based selective utilization. Finally, it outlines the ongoing challenges and future perspectives for the development and application of techniques for the multi-scale structural characterization of gluten proteins.

## 1. Introduction

Wheat stands as one of the world’s primary staple crops. According to the FAO Food Outlook released in November 2025 [1], global wheat production in 2025/26 is forecast to reach 819.2 million tonnes, accounting for one-third of global cereal consumption. Wheat flour-based products constitute a vital component of daily human diets, where the dough formation process plays a pivotal role in determining final product quality. Different wheat flour-based food products require flour with specific characteristics, among which gluten, the heterogeneous mixture of glutenins and gliadins serves, as the most critical one due to its serious impacts on dough formation.

**Figure 1:**
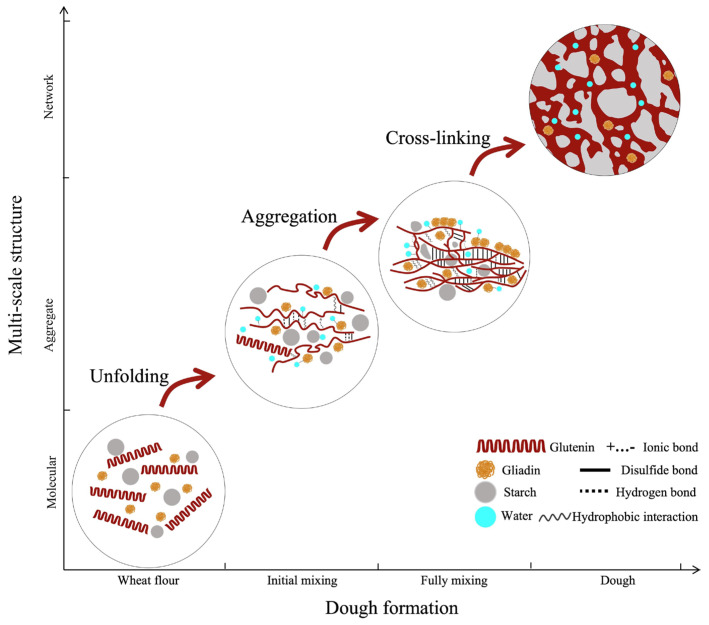
Proposed model for the development of gluten network (the changes in multi-scale structures) in dough formation.

Dough formation is defined as a dynamic process mainly consisting of three scales at molecules, aggregates, and networks of gluten proteins, respectively. As a consequence of this process, a three-dimensional network is constructed by gluten proteins via various intermolecular interactions with hydrated starch granules encapsulated in it. At the molecular scale, the hydration of gluten proteins initiates when the flour mixes with water, which is chiefly driven by the hydrogen bonds between the hydrophilic or charged groups of gluten proteins and water molecules. Upon hydration, gluten proteins undergo various structural transformations, such as the conversion of *β*-sheets into *β*-turns [2]. In the formation of gluten aggregates, the major structural evolution occurs with hydrated glutenins, which undergo sulfhydryl-disulfide interchange reactions and thus progressively assemble into more ordered threadlike structures, such as glutenin macropolymer (GMP) [3]. Finally, the above-mentioned gluten protein aggregates in turn interact with each other, presumably via hydrogen bonding, hydrophobic associations, and disulfide cross-linking, to form a three-dimensional network [4]. As this network develops, the dough’s viscoelasticity increases with kneading time, but overmixing can disrupt the structure and reduce its viscoelasticity. Unlike glutenins, gliadins interact with glutenins as monomeric spherical particles via non-covalent forces during dough making, and these interactions are traditionally considered to contribute to the viscosity of dough (Figure 1) [5,6].

In essence, the dough quality is ultimately determined by the formation of the above-mentioned multi-scale structures in gluten proteins. To evaluate the quality of a dough, it is necessary to discern the amount, structural features, and spatial distribution of these multi-scale structures in dough at different stages of dough formation. Currently, a large number of methods have been invented for this purpose; there are even several methods available for a certain object. For example, four methods can be used to evaluate changes in hydrophobic interactions during the dough formation process: fluorescence probe [7], acid-base indicator [8], molecular chromatography [9], and solubility method [4]. On the one hand, this offers a variety of method selections when designing a project according to the actual availability of instruments. On the other hand, this has resulted in a choice difficulty for researchers when multiple methods are available, as well as a difficulty for readers when comparing results from different methods. Bearing these problems in mind, the aims of this review focus on the following aspects: (1) systematically examining the characterization methods for the multi-scale structures of gluten proteins in dough; (2) discerning the pros and cons of different methods available for the same test indicator and clarifying the applicable scenarios for each method; (3) evaluating the working performance of a certain method when operated under different conditions and promoting their standardization; and (4) establishing an integrated methodological system for the gluten network in different types of dough or wheat flour-based products. This review will promote the technical progress in characterizing the multi-scale structures of gluten proteins and their evolution in dough formation.

## 2. Methods to Characterize Gluten Proteins at a Molecular Scale

### 2.1. Quantification of Gluten Fractions

Usually, gluten proteins in wheat can be divided into gliadins and glutenins on the basis of their solubility. Normally, gliadins and glutenins account for 50%–60% and 40–50% of gluten in wheat flour, respectively, although their proportions depend on the variety and origin of wheat [10,11]. The content and relative proportion of these fractions determine the processing quality of wheat flour. It is widely acknowledged that the dough elasticity and extensibility are attributed to gliadins, whereas the dough tensile strength is conferred by glutenins [12]. In this sense, the quantification of gluten fractions is the most fundamental work in characterizing the multi-scale structure of dough.

The quantification of gluten fractions always undergoes two steps: fractionation and quantification. As for the fractionation step, the methods of Osborne and Sequential extraction are often adopted [13,14]. Gluten proteins are fundamentally distinguished into two primary fractions (gliadins and glutenins) based on their differential solubility in specific solvent systems, a principle derived from the classic Osborne fractionation. This solubility-based separation is governed by the intricate interplay of molecular weight, surface hydrophobicity, and isoelectric points (pI) [15]. In the adapted Osborne procedure [13], non-gluten constituents (albumins and globulins) are first removed using water and dilute salt solutions to isolate the target matrix. The gliadin fraction is subsequently extracted using 70% ethanol; despite their relatively alkaline pIs (~7.8), their high surface hydrophobicity facilitates their selective solubilization as monomeric units in aqueous alcohol. The remaining glutenin fraction, characterized by pIs closer to 5.8 and the presence of large disulfide-linked aggregates, is then solubilized using dilute acid or alkali. This process promotes extraction by shifting the pH away from the glutenins’ pI, thereby increasing electrostatic repulsion to disrupt the polymeric assemblies.

Both methods can fractionate gluten proteins in wheat flour or dough into gliadins and glutenins (Supplementary File S1). In contrast, the Osborne method was invented earlier and applied more popularly than the Sequential extraction method, although both have their own advantages and disadvantages. Specifically, there were several more important aspects that differed between the two methods: (1) the Sequential extraction method demonstrated higher purities for all fractions than the Osborne method, especially a much lower contamination level of globulins/albumins in gliadins; (2) the Sequential extraction method achieved higher recoveries (up to 98.1%) for all fractions than the Osborne method; and (3) the Sequential extraction method required a higher solvent consumption per sample size than the Osborne method, which confers upon the former higher costs as well as lower environmental friendliness. Based on these, the Osborne method is well-suited for the large-scale preparation of gluten fractions, while the Sequential extraction method is particularly suitable for precise analysis of gluten sub-fractions, such as electrophoresis, chromatography, and proteomics.

Following the fractionation step, the subsequent quantification process is generally more straightforward. Commonly, the protein content in obtained gluten fractions is determined by the Kjeldahl nitrogen method [16], the Dumas combustion method [17], or the colorimetric method [18]. Among them, the Kjeldahl method always serves as a reference method. Both the Kjeldahl and Dumas methods are almost suitable for any case regardless of sample solubility, while the colorimetric method can be applied only to soluble proteins, although it always presents a lower limit of quantitation than the Kjeldahl and Dumas methods.

### 2.2. Analysis of Monomeric/Subunit Composition of Gluten Fractions

Gluten is a heterogeneous mixture of gliadins and glutenins. The gliadins are mainly subdivided into α/β-gliadin (25–35 kDa), γ-gliadin (35–40 kDa), and ω-gliadin (40–55 kDa) according to different mobility upon acid PAGE, with approximate proportions of 53%, 31%, and 16%, respectively [19]. For the glutenins, they are chiefly composed of low-molecular-weight (LMW-GS, 30–50 kDa) and high-molecular-weight (HMW-GS, 60–100 kDa) subunits with common proportions of 65–70% and 30–35%, respectively [20]. Furthermore, HMW-GS is usually divided into x-type (83–88 kDa) and y-type (67–74 kDa), while LMW-GS is often classified into B-type (42–51 kDa), C-type (31–37 kDa), and D-type (52–60 kDa) [5]. The common proportions of these subunit types are 65–75% (x) and 25–35% (y) in HMW-GS and 40–60% (B), 20–30% (C), and 5–10% (D) in LMW-GS, respectively. Both the composition of gliadin monomers and glutenin subunits depend on the cultivar and production area of wheat and ultimately confer significant impacts on dough formation [21]. Thus, this molecular information is valuable for clarifying the multi-scale structure of gluten during dough formation. Currently, electrophoresis, high-performance liquid chromatography, and proteomics are widely used to monitor the amounts and dynamic changes of these monomers, subunits, and types during dough formation.

#### 2.2.1. Electrophoresis

To identify and semi-quantitatively assess gliadin monomers and glutenin subunits, sodium dodecyl sulfate polyacrylamide gel electrophoresis (SDS-PAGE) and two-dimensional gel electrophoresis (2-DE) are often performed [22]. With respect to SDS-PAGE, SDS molecules bind to denatured proteins in a fixed ratio, effectively masking the inherent charge heterogeneity of protein molecules, resulting in a molecular size-directed separation [23]. After the operational procedures such as electrophoretic migration and staining [24,25], the monomers or subunits of proteins with significantly differing molecular weights stand as independent bands in the running gel, which allows their identity authentication, molecular weight measurement, and abundance determination via the locations and intensities (grayscale) of these bands by referring to standard markers (Supplementary File S2). To obtain only the information on monomer and subunit/type composition, a reducing agent such as *β*-mercaptoethanol or dithiothreitol is used, which can cleave disulfide bonds and thereby depolymerize any monomer/subunit aggregates [26]. In this case, by comparing the results with (reduced SDS-PAGE) or without (non-reduced SDS-PAGE) a reducing agent, the details of the aggregate formation among monomers or subunits via disulfide linkages can be clearly revealed [27].

In regard to 2-DE, it includes two sequentially arranged separation processes in different dimensions: the first dimension is realized by isoelectric focusing, which allows the satisfactory separation of protein molecules according to their isoelectric points, and in the second dimension, SDS-PAGE is performed, which is perpendicular to the first dimension and further separates protein molecules in light of their molecular weight [28]. Usually, 2-DE is performed in a reduced mode with the utilization of DTT to ensure that protein molecules are completely denatured and remain reduced [29]. Under these circumstances, a high-resolution 2D protein map is concluded from 2-DE, which enables a clear resolution of monomers or subunits in gluten fractions as numerous discrete spots (Supplementary File S3). As in SDS-PAGE, the identity authentication, molecular weight measurement, and abundance determination of these spots can be accomplished by checking their locations and intensities (grayscale). Moreover, the identification of protein spots could be fulfilled with mass spectrometry.

Based on the above discussions, both SDS-PAGE and 2-DE have their respective advantages and limitations, which can be mainly summarized in the following two aspects. Firstly, SDS-PAGE is more popular than 2-DE due to easier operation, lower cost, higher throughput, and better time savings. The operating conditions of 2-DE are more stringent than those of SDS-PAGE, such as lower temperature (~4 °C/room temperature), greater variety of chemicals (for isoelectric focusing), higher voltage (˃60 kV/˂150 V), and more restricted reduction with DTT and iodoacetamide [28]. For one experimental cycle, typical 2-DE can only handle one sample per gel and often consumes 6–8 h, while SDS-PAGE can manage up to ten samples per gel and complete an analysis cycle within 2–4 h. Secondly, 2-DE is significantly superior to SDS-PAGE in resolution and sensitivity. Unlike 2-DE, SDS-PAGE cannot resolve proteins with identical molecular weights but different origins or types [30]. In addition, the sensitivity of SDS-PAGE is insufficient for detecting low-abundance proteins. 2-DE is capable of detecting over 1000 protein spots per gel at a nanogram detection limit, while SDS-PAGE can only accommodate up to tens of bands within each lane at a microgram detection limit [31]. In summary, to understand the insights of monomeric or subunit components in gluten fractions, SDS-PAGE is suitable for the fractions with fewer components, and 2-DE is valuable in cases where a comprehensive fraction with a large number of components is under consideration.

#### 2.2.2. High-Performance Liquid Chromatography

To separate and identify the protein components in a certain gluten fraction, size-exclusion high-performance liquid chromatography (SE-HPLC) and reversed-phase high-performance liquid chromatography (RP-HPLC) are sometimes applied, although they work in different modes [32]. SE-HPLC separates protein targets based on their molecular weight or, more specifically, hydrodynamic volume. As a result, a size-based elution curve is produced, in which protein components are arranged in order of molecular weight against the elution time. The larger the size of a component, the earlier the location it occupies (Supplementary File S4). Based on the retention time and area of an elution peak, the protein component can be identified, and its molecular weight and abundance can be estimated [33]. Similar to electrophoresis, SE-HPLC can be performed either in reduced mode or non-reduced mode [34]. For this method, the complete dissolution of the gluten fraction is essential to obtain satisfactory accuracy and reproducibility. To this end, ethanol (~50%), SDS (~1%), and urea (~6 M), even at high concentrations, were used to dissolve samples [35]. Even though the performance of SE-HPLC is usually not satisfactory in analyzing the components of the gluten fraction, the failure is evident in its inability to distinguish between the different types of HMW-GS and LMW-GS, as well as in its suboptimal resolution for gliadin monomers [36].

As for RP-HPLC, it separates proteins based on their differences in hydrophobicity, in which the elution orders of protein components are determined by the intensities of their hydrophobic interactions with the non-polar stationary phase [37]. With a specific column, the higher the hydrophobicity of a protein component, the later it elutes from the column. In such cases, a hydrophobicity-based elution curve was concluded for all protein components against the elution time [38]. The protein corresponding to each elution peak can be identified with retention time, and its abundance can be estimated with peak area. Previous results showed that RP-HPLC can effectively resolve gliadin monomers and the x-type and y-type subunits of HMW-GS [39,40], but it is unable to distinguish the B-type, C-type, and D-type subunits within LMW-GS (Supplementary File S5).

As SE-HPLC and RP-HPLC were compared, they differed from each other in the following aspects: (1) they obey different working principles, and thus the protein components were eluted in different sequences; (2) SE-HPLC could provide information on the identification, molecular weight, and abundance of components, while RP-HPLC was only informative regarding component identification and their abundance; (3) RP-HPLC has much higher resolution than SE-HPLC, although both of them are more effective in discriminating gliadin monomers than glutenin subunits; and (4) in view of operation mode, SE-HPLC always adopts isocratic elution, while RP-HPLC requires gradient elution, which suggests that the efficacy of RP-HPLC demonstrates a higher dependence on operational conditions than that of SE-HPLC [41]. Based on these, SE-HPLC is recommended to acquire the information on molecular weight distribution of gluten fractions, especially the gliadin fraction, while RP-HPLC can be applied to identify and quantify the monomers and subunits in gluten fractions.

#### 2.2.3. Proteomics

**Figure 2:**
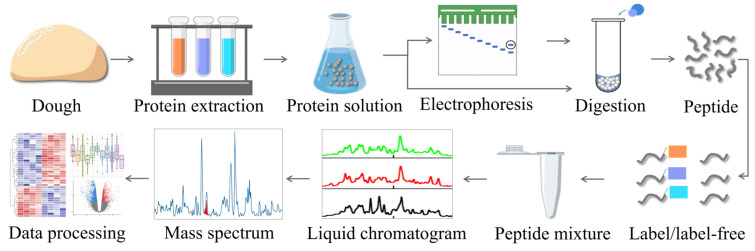
The workflow of mass spectrum (MS)-based proteomics.

In recent decades, mass spectrometry (MS)-based proteomics has achieved significant developments and successfully evolved into a comprehensive analytical workflow (Supplementary File S6) for large-scale identification and precise quantification thousands of proteins from complex samples, such as gluten or its fractions. Generally, the proteomic workflow chiefly consists of the following procedures: (1) sample preparation; (2) protein extraction, separation, and digestion; (3) peptide separation and mass analysis; and (4) data analysis (Figure 2) [42,43]. Moreover, 2-DE for protein separation, the other crucial techniques applied in proteomics often include stable isotope labeling of proteins and peptides, high-resolution liquid chromatography (mainly RP-HPLC), and targeted and discovery-type MS, as well as tailored signal processing algorithms, database searching tools, and downstream biostatistics.

In sample preparation, a defatting procedure is necessary for wheat flour or dough samples to eliminate lipid interferences [44]. The details on protein extraction were discussed above in Section 2.1. Furthermore, the obtained gluten or gluten fractions undergo an enzymatic digestion process to be converted into peptides with a size ranging from 6 to 20 amino acid residues. For high-throughput global proteomic analysis (shotgun proteomics), an in-solution enzymatic digestion is directly implemented with extracted proteins without protein separation [45]. Alternatively, an in-gel enzymatic digestion is often used for each spot resulting from 2-DE of extracted proteins [29]. In contrast to in-gel digestion, in-solution digestion is suitable for high-throughput and comprehensive profiling of protein composition, but due to peptide mixture complexity and shared sequences among homologous proteins, it suffers from ambiguities in protein monomer and subunit inference. Typically, in common proteomics, trypsin is used in digestion. However, in the digestion of gluten or gluten fractions, chymotrypsin is often employed to complement trypsin to achieve enough digestion efficiency and peptide coverage, due to the high presence of glutamine and proline residues in gluten proteins [23]. Prior to peptide separation, the digests are often subjected to a peptide purification procedure to remove interfering substances, such as salts, denaturants, elution agents, and gel residues, and thus achieving a high peptide recovery and thereby ensuring the sensitivity and accuracy of mass analysis. This is typically achieved via solid-phase extraction for large-volume specimens from in-solution enzymatic digestion and ZipTip C18 microcolumns for small-volume specimens from in-gel enzymatic digestion [46].

For peptide separation and mass analysis, they are often performed in an integrated mode using advanced analytical platforms, such as LC-MS/MS, NanoLC-Orbitrap-MS/MS, and NanoLC-ESI-QqTOF-MS/MS, to first finely resolve and then structurally characterize the peptides in purified digests [47]. It must be noted that both label-free and stable isotope labeling techniques are applied for mass analysis. In contrast, isobaric labeling techniques such as isobaric tags for relative and absolute quantification and tandem mass tags allow improved precision [48]. Data analysis is performed using dedicated software platforms [49], e.g., MaxQuant version 1.6.0.1, Proteome Discoverer version 3.1, and Mascot version 2.1, which support peptide identification, protein quantification, and functional annotation.

Compared to its sister techniques of electrophoresis and RP-HPLC as mentioned above, proteomics demonstrates much higher sensitivity and resolution. The detection limits of protein components are as low as picomolar levels, and it can quantify protein components at concentrations below 1 ng [50], thus delivering more accurate qualitative and quantitative data. Moreover, it also provides broader proteome coverage, identifying up to 3000–5000 proteins in complex protein systems. These advantages make proteomics an indispensable tool in analyzing the monomeric/subunit composition of gluten fractions, particularly for characterizing low-abundance gluten components or when the information of detected components is unavailable in the protein database. Nonetheless, the application of proteomics remains constrained by complex sample preparation, high instrumentation costs, challenging data analysis, and reliance on protein sequence databases.

### 2.3. Structural Characterization of Gluten Molecules

As discussed above, the primary structure of gluten proteins can be elucidated through proteomics. Other structural information at the molecular level of gluten proteins includes their secondary structures as well as the bonds/groups maintaining or narrating their tertiary and even quaternary structures, mainly including the content of sulfhydryl (SH) and disulfide bonds (SS), the conformation of SS, and the microenvironment of aromatic amino acids.

#### 2.3.1. Secondary Structure

As in other proteins, the secondary structural elements in gluten molecules mainly include *α*-helices, *β*-sheets, *β*-turns, and random coils. Among them, *α*-helices, *β*-sheets, and *β*-turns represent stable and ordered conformations, while random coils are intrinsically disordered structures [51]. For the measurement of secondary structures of gluten proteins extracted from dough, Fourier transform infrared spectroscopy (FT-IR) and Fourier transform Raman spectroscopy (FT-Raman) are often applied.

Both FT-IR and FT-Raman provide two regions of the resulting spectra are very informative in quantifying the relative abundances of secondary structural elements: amide I (1700–1600 cm^−1^) and amide III (1220–1330 cm^−1^) [52]. In contrast, the amide I band is more widely used due to its stronger signal and higher sensitivity. However, the amide III band is less susceptible to water interference in FT-IR and contains fewer peak overlaps, thus offering superior spectral resolution compared to amide I, although it has a much weaker signal intensity (about 1/5 of amide I) [53]. In light of this, amide III allows for a clearer identification of secondary structural elements and is more suitable for hydrated samples in FT-IR than amide I.

For both amide I and amide III, a typical workflow of FT-IR and FT-Raman involves spectrum acquisition, spectrum processing, band assignment, and data calculation (Supplementary Files S7 and S8) [54]. Among them, spectrum processing and band assignment are of higher importance in determining analysis quality. As for spectrum processing, it always includes water subtraction (only for FT-IR), baseline correction, Gaussian smoothing, normalization, second-derivative analysis, and Fourier self-deconvolution [55]. For the water subtraction with FT-IR, the subtraction of the water spectrum at the moisture level of the sample by using OMNIC 8.0 software is executed to eliminate the interference of water molecules present in samples, whose absorption bands in both amide I and amide III certainly interfere with those from proteins [56]. Alternatively, the interference of water molecules could be avoided by substituting water with deuterium oxide in dough formation [51]. The other processing steps, except for water subtraction, can be performed using the corresponding functions provided in the OMNIC software. Baseline correction, Gaussian smoothing, and normalization can prevent the appearance of side oscillation artifacts, attenuate the influence of noise, and eliminate differences in sample amounts, respectively. Second-derivative analysis and Fourier self-deconvolution address the challenge of spectral overlap [27]. Usually, the assignment of the resulting resolved bands is fulfilled by referring to the data from the literature (Table 1) [57]. As for curve fitting, it is often implemented by the PeakFit v4.12 software. As a result, the best fits are achieved for the well-resolved peaks from second-derivative analysis and deconvolution, which allows for the accurate evaluation of their areas. Finally, the percentage of specific secondary structure is calculated by dividing the assigned band area by the total area of all secondary structures [52].

As mentioned above, FT-IR and FT-Raman highly resemble each other in analyzing the secondary structures of gluten and gluten fractions, but differ from each other in the following aspects [58]: (1) they obey different working principles, i.e., FT-IR is based on the absorption of infrared radiation, while FT-Raman relies on the frequency shifts in scattered laser light due to inelastic scattering processes [59]; (2) FT-IR is susceptible to the moisture level in tested samples, while FT-Raman is of intrinsic resistance to water interference; and (3) FT-IR spectroscopy offers higher sensitivity, stronger signal intensity, and quicker data acquisition than FT-Raman [60]. To guarantee enough signal intensity for detection, FT-Raman generally requires a longer acquisition time or a higher sample concentration than FT-IR. Thus, FT-IR is superior to FT-Raman in high-throughput screening of dried solid samples, such as freeze-dried dough powders, while FT-Raman is more effective than FT-IR in working with hydrated or aqueous systems, such as fresh dough pieces. Due to these facts, FT-IR is more popular than FT-Raman in measuring the relative abundance of the secondary structures in gluten.

**Table 1:** Fourier transform infrared spectroscopy (FT-IR) and Fourier transform Raman spectroscopy (FT-Raman) characteristic absorption peaks for protein secondary structures and disulfide bond vibrations (cm^−1^).

Method	Amide Region	*β*-Sheet	Random Coils	*α*-Helix	*β*-Turn	References
FT-IR	Amide I	1600–1640 1685–1700	1640–1650	1650–1660	1660–1685	[55,57]
	Amide III	1220–1245	1255–1270	1295–1330	1270–1295	[52]
FT-Raman	Amide I	1612–1640	1660–1665	1650–1658	1655–1675	[59]
	Amide III	1229–1235	1243–1253	1270–1300	1265–1286	[59,61]

#### 2.3.2. Tertiary and Quaternary Structures

##### The Content of SH and SS

Due to their richness in cysteine residues (2%) [11], gluten proteins contain a large number of SH groups, which often undergo interchain or intrachain disulfide exchange reactions upon oxidation to be converted into disulfide bonds [62]. Thus, this gives rise to more SS in gluten proteins upon the consumption of SH. As a consequence, the conversion of SH into SS could either promote the folding of peptide chains or establish new cross-linking between peptide chains. This, especially the formation of interchain and intramolecular disulfide bonds, enhances the stability of tertiary and quaternary structures by decreasing the configurational entropy of polypeptide chains [63].

The amount of SH (*F*_-SH_) is typically determined directly in samples using Ellman’s reagent by a spectrophotometric method (Supplementary File S9) at 412 nm [55]. However, to determine the amount of SS, the sample is first subjected to a reduction to completely convert SS into SH, and then total SH (*T*_-SH_) is determined. The amount of SS is calculated as half the difference between *F*_-SH_ and *T*_-SH_. To reduce SS, *β*-mercaptoethanol (*β*-ME) and tris (2-carboxyethyl) phosphine (TCEP) methods are often applied [64], but they differ in the following aspects: (1) the *β*-ME method is more popular than the TCEP method due to its longer usage history and lower cost; (2) the *β*-ME method is safer, more stable, and user-friendly than the TCEP method because *β*-ME is volatile, malodorous, and prone to oxidation, while TCEP is odorless, nonvolatile, and resistant to air oxidation; (3) the *β*-ME method demonstrates a greater background interference and a lower measurement accuracy than the TCEP (without SH) method in light of the fact that, as a thiol-bearing agent, the unreacted *β*-ME largely leads to an overestimation of total SH and SS; (4) in terms of the time required for the reduction, *β*-ME method (approximately 150 min) is more time-consuming than the TCEP method (around 25 min) [8]; and (5) the introduction of urea and guanidine hydrochloride makes the *β*-ME method harsher than the TCEP method. Taking these into consideration, the *β*-ME method is well-suited for large-scale measurements at a lower cost, while the TCEP method is more effective for precise measurements and for measurements that may better preserve the native conformation of targeted proteins due to its milder reducing conditions.

##### The Conformation of SS Bonds

Besides its content, the conformations of disulfide bonds play a crucial role in maintaining the folding and stability of proteins. In general, the conformations of disulfide bonds are defined by the orientations of the C-S-S-C bridge, and thus disulfide bonds are accordingly categorized into rotational isomers, namely, gauche-gauche-gauche (SS*_g-g-g_*), trans-gauche-gauche (SS*_t-g-g_*), and trans-gauche-trans (SS*_t-g-t_*) [65]. Among them, the SS*_g-g-g_* conformation is typically stable and helps maintain the correct tertiary structure of proteins. In contrast, SS*_t-g-g_* and SS*_t-g-t_* conformations are generally unstable, and an excess of these conformations suggests that the protein may be in an abnormal folding state and have an unstable tertiary structure [63]. This instability may result from disordered polypeptide chain displacement caused by abnormal folding and subunit aggregation of the proteins. In this sense, the elucidation of the disulfide bond’s conformation is helpful in understanding the tertiary structures of proteins. To quantify the relative abundances of these three isomers, the FT-Raman spectrum is always adopted, in which the region of 500–550 cm^−1^ can provide valuable information [66]. The bands across the wavenumber ranges of 500–510 cm^−1^, 515–525 cm^−1^, and 535–545 cm^−1^, peaking at 505 cm^−1^, 520 cm^−1^, and 530 cm^−1^, are assigned to SS*_g-g-g_*, SS*_t-g-g_*, and SS*_t-g-t_*, respectively. Regarding the operational details for FT-Raman, it is exactly the same as that done in the measurement of the secondary structures.

##### The Microenvironment of Aromatic Amino Acids

As is known to all, the exposure or burial of certain amino acid residues depends on the changes in the tertiary and quaternary structures of proteins. When an amino acid residue is exposed or buried, it poses different microenvironments. Thus, conversely, it is possible to understand the tertiary and quaternary structures of proteins by exploring the microenvironments of certain amino acid residues. Usually, aromatic amino acid residues, mainly tyrosine (Tyr) and tryptophan (Trp), are under consideration due to their easy detectability and sensitivity to the changes in the advanced structures of proteins. To detect the microenvironments of these residues, FT-Raman and fluorescence spectroscopy are widely used [67].

As for FT-Raman spectroscopy, the microenvironments of Tyr and Trp are often determined with the spectrum being normalized against the band of phenylalanine (Phe), whose intensity and location are not sensitive to protein conformation [68]. In FT-Raman spectroscopy, Tyr residue shows two characteristic bands at 830 cm^−1^ and 850 cm^−1^, while Trp presents only one at 760 cm^−1^. In measuring their microenvironments, doublet value (*I*_850_/*I*_830_) and peak intensity (*I*_760_) are used for Tyr and Trp, respectively. Usually, an increase in *I*_850_/*I*_830_ or a decrease in *I*_760_ implies the protein achieves more unfolded advanced structures. Conversely, a decrease in *I*_850_/*I*_830_ or an increase in *I*_760_ suggests that more compact advanced structures are attained by the protein. Commonly, the doublet values of exposed, normal, and buried Tyr residues fall in the ranges of >1.50, 0.90–1.43, and 0.30–0.90, which indicate loosened (unfolded), normal, and compacted (folded) conformations of tested proteins, respectively [67].

For fluorescence spectroscopy, it probes the tertiary and quaternary structures of proteins by mainly measuring the intrinsic fluorescence of Trp residue, due to its dominant contribution to the total fluorescence of protein and high sensitivity to microenvironment polarity [69]. Typically, Trp residues present the maximum emission peak around 350 nm. It is found that the position and fluorescence intensity of this peak highly correlate with the states of advanced structures of protein [70]. A shift toward lower wavelengths (blue shift) with an increase in peak intensity indicates a more compact structure, whereas a shift toward longer wavelengths (red shift) with a decrease in peak intensity reflects that the protein undergoes loosening or unfolding.

In elucidating the tertiary and quaternary structures of gluten proteins, the pros and cons of FT-Raman spectroscopy and fluorescence spectroscopy lie in the following aspects: (1) in terms of operational easiness (Supplementary File S10), two methods are comparative due to the fact that the FT-Raman method requires a complicated spectral processing, while in the fluorescence method, a protein extraction process is necessary [71]; (2) the fluorescence method is superior to FT-Raman method in detection sensitivity, which allows the measurements in the cases with extremely low presence of Trp residues (pmol~nmol); (3) the FT-Raman method offers chemical-free non-destructive analysis with sample states in solid, hydrated, or turbid, whereas the fluorescence method is destructive, requiring protein extraction and the preparation of transparent solutions with chemicals; (4) the conditions of samples, such as pH and temperature impose a greater influence on the fluorescence method than the FT-Raman method, mainly due to their effects on protein extraction; and (5) both methods are susceptible to the interference from sample autofluorescence, but this is particularly serious in the fluorescence method when it works at a high protein concentrations (>0.5–1 mg/mL) [69], in which self-quenching and inner filter effects are markedly enhanced. In summary, the fluorescence method is recommended in cases where only a small amount of sample is available or a high analysis accuracy is needed, while the FT-Raman method is better suited for online non-destructive monitoring or the comparison of samples with significantly different conditions, such as pH.

## 3. Methods to Characterize Gluten Proteins at an Aggregate Scale

### 3.1. Analysis of Aggregation Degree

#### 3.1.1. The Degree of Overall Aggregation

**Figure 3:**
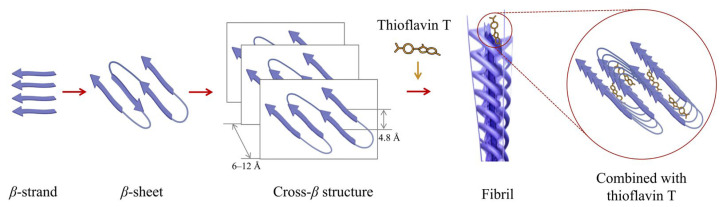
Schematic diagram of the formation of *β*-structured fibrils and their binding processes with thioflavin T.

The previous research has revealed that the aggregation of protein molecules always results in the formation of fibrillar *β*-sheet structures [72,73]. They are formed through the following steps (Figure 3): (1) the strong but non-covalent interactions occur between *β*-sheets from different protein molecules; (2) the interactions of *β*-sheets give rise to the stacking of protein molecules into protofibrils; and (3) the resulting protofibrils subsequently assemble into fibrillar *β*-sheet structures, i.e., large fibrils with a diameter of several nanometers and a length up to many microns, which possess a characteristic cross-*β* conformation. As a result, the amount of fibrillar *β*-sheet structures is indicative of the overall aggregation degree of protein molecules. To quantify this, a front-face fluorescence method using the probe of Thioflavin T [26,74] is commonly used. In this method, Thioflavin T can insert into the groove sites of fibrillar *β*-sheet structures and results in both excitation (approximately from 336 nm to 435 nm) and emission (approximately from 445 nm to 482 nm) shifts. These shifts are absent from the interaction of Thioflavin T with the monomers/subunits and small oligomers of gluten proteins, as well as the aggregates of gluten proteins lacking cross-*β* structure. Thus, the overall aggregation degree of proteins or the amount of fibrillar *β*-sheet structures could be quantified by measuring the maximum fluorescence intensity at 482 nm with excitation at 435 nm (Supplementary File S11). This method is highly sensitive, capable of detecting early-stage, low-concentration aggregates, and can be applied to in situ detection of real food samples, including hydrated dough and prepared flour-based products. However, it has certain limitations. First, it is incapable of distinguishing the types of aggregates from different gluten subunits, e.g., glutenins and gliadins. Second, its test results are greatly influenced by environmental factors such as pH and ionic strength. Third, a serious background interference happens at high concentrations of gluten proteins.

#### 3.1.2. The Degrees of Covalent and Non-Covalent Aggregations

In essence, the formation and survival of gluten aggregates are driven by the intermolecular forces among gluten proteins, including covalent and non-covalent forces [75]. The covalent force refers to disulfide bonds, and the non-covalent forces include hydrophobic interactions, electrostatic interactions, and hydrogen bonds. Based on this, the overall aggregation of gluten proteins could be further resolved into covalent and non-covalent components. As these names imply, the degrees of covalent and non-covalent aggregations are defined as the contribution ratios of covalent and non-covalent bonds in the formation and stabilization of gluten aggregates. Disulfide bonds remain stable, but non-covalent bonds are liable to be disrupted in the presence of SDS. When the driving forces are disrupted, the gluten aggregate undergoes disintegration, and the resulting monomers/subunits become soluble in an SDS solution. Hence, the non-covalent component could be resolved from the covalent one by determining the solubility of gluten aggregates in a SDS solution [76], typically at a concentration of 0.5% in phosphate buffer (pH 7.0). Conversely, the amount of SDS-insoluble protein represents the covalent component [77]. In greater detail, the non-covalent component could be subdivided into strong, medium, and weak non-covalent fractions by using different concentrations of SDS solution as 0.5%, 0.3%, and 0.1%, respectively (Supplementary File S12). Fundamentally, the strong, medium, and weak non-covalent fractions are certainly attributed to the non-covalent bonds with different strengths. The strong fraction results from strong hydrogen bonds and strong ionic bonds, the medium fraction relates to strong hydrophobic interactions, weak hydrogen bonds, and weak ionic bonds, while the weak fraction depends on weak hydrophobic interactions. This method is of easy operability and good reproducibility, but it cannot achieve a complete resolution of the covalent component from the non-covalent one because the non-covalent bonds certainly remain in SDS-insoluble aggregates, although they are chiefly stabilized by disulfide bonds.

### 3.2. Analysis of Gluten Aggregates

To accurately analyze the appearance (size and shape) of gluten aggregates, a workflow encompassing controlled extraction, size-based fractionation, and precise quantification is required.

#### 3.2.1. Controlled Extraction

For controlled extraction, the key point is to obtain gluten aggregate suspension with minimal intervention to their native appearances. It often employs a mild neutral buffer system free from strong denaturants such as SDS or urea. A typical controlled extraction is implemented at a low temperature (<20 °C) by using 50% (*v*/*v*) aqueous ethanol as the extractant [36]. To explore the contributions of various covalent and non-covalent bonds to the formation of gluten aggregates with different sizes, the extractants capable of selectively disrupting specific bonds are applied. Specifically, to investigate the contributions of weak hydrophobic interactions, strong hydrophobic interactions, hydrogen bonds, and electrostatic interactions, 10% (*v*/*v*) aqueous 1-propanol, 30% (*v*/*v*) aqueous 1-propanol, 4 M urea, and 0.1–0.6 M NaCl or 0.05–0.1 M acetic acid (pH 4.5) are applied [26,78], respectively. To investigate the contribution of disulfide bonds, the extraction should be carried out in both reducing and non-reducing modes by using the extractants of PBS containing 2% (*w*/*v*) SDS and 1% (*w*/*v*) DTT, *β*-ME, or TCEP, respectively [32]. The difference between the two conditions represents the contribution of disulfide bonds.

#### 3.2.2. Size-Based Fractionation

**Figure 4:**
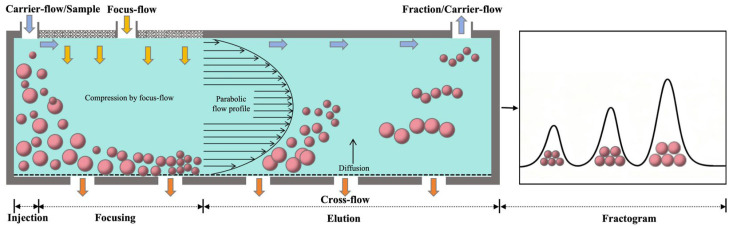
Schematic diagram for the operation of asymmetrical flow field-flow fractionation.

For size-based fractionation, the extracted gluten aggregates are often subjected to size-exclusion chromatography (SEC) or asymmetrical flow field-flow fractionation (AsFlFFF), which separates gluten aggregates as consecutive eluents according to their physical sizes [79]. The working law and operation highly resemble those of SE-HPLC as discussed above in Section 2.2.2. As for AsFlFFF, it is based on the differences in Brownian diffusion capability or flow velocity of size-different particles in a velocity-gradient laminar flow field [80]. To perform this, an Eclipse AsFlFFF system is often used, in which the particle fractionation occurs in an asymmetrical thin channel without a stationary phase (Supplementary File S13). When the mobile phase is flowing through the channel, a laminar flow field is thus generated, with the flow rate increasing from the bottom to the middle of the channel. In this field, the smaller particles are more liable to migrate against the velocity gradient of the field due to their higher diffusion coefficients. This propels the smaller particles to migrate into the middle faster flow lines while the larger ones remain in the bottom slower flow lines. Thus, the smaller the particle, the higher the flow rate it attains. As a consequence, the particles elute in an order from smallest to largest in size from the channel (Figure 4). To improve the resolution, the new system jointly applies focus flow, cross flow, and carrier flow in injection, focusing, and elution programs to precisely monitor the locations and flow velocities of these particles in the channel [81].

The advantages and disadvantages of SEC and AsFlFFF [82,83] can be summarized in the following aspects: (1) in contrast to AsFlFFF, SEC is more mature and presents better performance in low cost, easy operability, quick analysis, and high reproducibility; (2) SEC demonstrates a stronger disruption to gluten aggregates than AsFlFFF due to the shear/rubbing effects generated when the particles travel through the voids/gaps of its stationary phase, namely, the porous gel particles; and (3) for the samples, both SEC and AsFlFFF are applicable, the former often providing a higher resolution than the latter, though AsFlFFF (~11,000 nm) can successfully resolve particles with a much broader size range than SEC (~5–100 nm). Taking these into account, SEC is preferentially recommended if the size range of gluten aggregates is located within its limits, while AsFlFFF is particularly suitable for proteiform or unstable aggregates, as well as for the samples with an unknown particle size range or containing ultra-large size gluten aggregates.

#### 3.2.3. Precise Quantification

To characterize the size-different components of gluten aggregates, a profile of consecutive eluates from SEC or AsFlFFF is recorded against elution time. By analyzing this profile, the mass, size, molecular weight, and shape of each component can be concluded. According to the purpose of the test, the profile is often recorded by selectively using one or more concentration-sensitive detectors, such as ultraviolet absorbance (UV), differential refractive index (DRI), multi-angle light scattering (MALS), and dynamic light scattering (DLS). SEC/AsFlFFF-UV/DRI-DLS-MALS [84,85,86] setups possess the most comprehensive functions. Notably, these detectors have different detection capabilities [37,79,87].

Among them, UV and DRI are selected when there is a need to determine the concentration of size-fractionated components as well as their mass and relative molecular weight. The UV detector operates based on the strong ultraviolet absorption of aromatic amino acid residues in gluten at approximately 280 nm, where the absorption intensity is linearly proportional to the protein concentration. DRI is based on the refractive index difference between the solution and the pure eluent, with this difference being proportional to the sample concentration. As the two most commonly used concentration detectors, UV and DRI differ in the following aspects [88]: (1) UV is more popular and easier to operate than DRI; (2) the signals from UV highly depend on both the density of UV-absorbing chromophores and the mass of tested proteins, which certainly impairs the comparability of the outcomes from different proteins. Definitely, this impairment is free from DRI. Thus, DRI is more applicable and accurate than UV in certain scenarios, such as comparing samples from different proteins with significantly different UV-absorbing capacities and analyzing samples with weak UV-absorbing capacity. On the contrary, the UV absorption dependence makes UV much more resistant than DRI against the interferences from substances without ultraviolet absorption, such as polysaccharides and lipids; and (3) UV is of a higher concentration sensitivity than DRI, and their linear detection ranges are assumed to be ~0.1–300 µg/mL and ~0.1–25 mg/mL, respectively. In the light of these points, UV offers better applicability to samples with low protein levels or high non-protein contaminants, while DRI is highly recommended when the aggregates exhibit a weak UV-absorbing capacity or the samples from different protein sources are under comparison.

DLS is selected when the research focus is on the size scale and the uniformity of the size distribution of the gluten aggregates. It can provide size scale in terms of hydrodynamic radius (*R*_h_) and polydispersity index (*PDI*, based on *R*_h_) [36]. This technique functions by monitoring the fluctuations in scattered light intensity caused by the Brownian motion of particles. Because the rate of these fluctuations is inversely related to particle size, it allows for the calculation of *R_h_*, which represents the effective size of the aggregate as it diffuses in the solvent, via the Stokes–Einstein equation. Furthermore, the *PDI* is determined by analyzing the decay curve of the light scattering autocorrelation function, as the distribution of decay rates reflects the overall breadth of the size distribution. DLS offers exceptional sensitivity and supports online real-time monitoring; however, its intensity-weighted nature in polydisperse gluten systems often allows trace large aggregates to mask predominant small-molecule components. Additionally, algorithms based on spherical assumptions lead to inherent biases when characterizing non-spherical aggregates. To resolve these discrepancies, the size scale in radius of gyration (*R*_g_) from MALS should be incorporated to rectify conformational deviations via the *R*_g_*/R*_h_ ratio [36], alongside cross-validating DLS intensity distributions with UV/DRI mass-weighted distributions [85]. This multi-detector coupling strategy effectively enhances the accuracy and reliability of gluten characterization.

The principle of MALS involves measuring the intensity of light scattered by a sample at multiple angles simultaneously. This angular dependence allows for the determination of absolute molecular weight (*M*), *R*_g_, and particle interaction intensity as characterized by the second virial coefficient. In terms of *M*, the aggregation degree could be evaluated. The monomeric proteins, protein aggregates, and ultra-large size protein aggregates are often evidenced within the *M* ranges of 3 × 10^4^–10^5^ g/mol, 10^5^–2 × 10^6^ g/mol, and >2 × 10^6^ g/mol, respectively. In the light of the equation of *R*_g_ = *k* × *M^α^*, the scaling exponent *α* is indicative of the shape of aggregates [89], namely, compact sphere (*α* ≈ 0.33), random coil (0.4 < *α* < 0.6), and rod-like (*α* ≈ 1.0). Moreover, the positive, zero, and negative values of the second virial coefficient indicate the interaction types and intensities between aggregates of repulsive, weak, and attractive nature, respectively [90]. The core advantage of MALS lies in its capability to determine absolute molecular weight and conformation without the need for column calibration with standards. However, its application is constrained by the requirement for sufficiently dilute conditions to avoid multiple scattering effects.

In addition, the shape of gluten aggregates can also be characterized by jointly using DLS and MALS, because the derivative parameter of the *R*_g_*/R*_h_ ratio is aggregate shape dependent. The *R*_g_/*R*_h_ ratio values of 0.77, 0.778, 1.0, 1.33, 1.50, 1.73, and >2.0 suggest the aggregates are in the shape of a hard sphere, homogeneous sphere, hollow sphere, regular star, monodisperse random coil, polydisperse random coil, and rigid rod, respectively [91,92]. Moreover, the detector combination of MALS-UV/DRI can measure number average molecular weight (*M*_n_) and weight average molecular weight (*M*_w_), thus further narrating the molecular weight distribution of aggregates in terms of molecular weight dispersity (*M*_w_*/M*_n_).

### 3.3. Analysis of the Bonds Involved in Gluten Aggregates

As discussed above, the gluten aggregates are maintained by various covalent and non-covalent bonds, including disulfide bonds, hydrophobic interactions, electrostatic interactions, and hydrogen bonds. The quantification of these bonds is of extreme importance in elucidating the precise structure of gluten aggregates. The measurement of disulfide bonds has been detailed in Section The Content of SH and SS; thus, only the quantification of non-covalent bonds is discussed in this section.

To determine the levels of non-covalent bonds, different solvents are often employed to selectively dissociate specific non-covalent bonds. As a consequence, the dissociation of non-covalent bonds causes an increase in protein solubility. Thus, the solubility increment upon the dissociation of a certain non-covalent bond could represent its level in gluten aggregates. In practice, two regimes, cascade and parallel, are adopted, although they differ in result calculation [4,17]. The former only uses one sample specimen, and the different solvents are supplied in succession, while the latter requires three identical sample specimens, and the measurements are performed in solvent-specimen couples (Supplementary File S14). For the cascade regime, the solvents are always used consecutively to dissociate the non-covalent bonds in a sequence of electrostatic interactions > hydrogen bonds > hydrophobic interactions. Corresponding to this sequence, the typical solvents include 0.6 M NaCl, 0.6 M NaCl containing 1.5 M urea, and 0.6 M NaCl containing 8 M urea. In contrast to the parallel regime, the cascade regime consumes a smaller quantity of samples but has a longer testing duration.

## 4. Methods to Characterize Gluten Proteins at a Network Scale

The gluten network is the most sophisticated and largest assembly of gluten proteins formed in prepared dough, which is a consequence of the covalent and non-covalent interactions among gluten aggregates as mentioned above. In the formation of the gluten network, the above-mentioned threadlike gluten aggregates first undergo interaction and interpenetration to form larger gluten aggregates, called gluten sheets. Then, the sheets layer one over the other and cross-link with each other to result in a gluten network with a three-dimensional structure [93]. As a consequence, the gluten network is a nanoporous ultrastructure that serves as the continuous phase and skeleton of dough with the dispersion phase, mainly starch granules, entrapped in it [94]. The microstructural characterization (e.g., junction density, lacunarity, and branching rate) of the gluten network is of extreme importance because its distinctive features provide dough with a unique viscoelasticity, granting the production of various wheat-based foods and determining their intermediate and final quality. To this end, various microscopic imaging techniques are employed, such as confocal laser scanning microscopy (CLSM), confocal Raman microscopy (CRM), scanning electron microscopy (SEM), and two-photon excitation microscopy (2PEM). Notably, these techniques vary in terms of their detection capabilities, such as the mode of operation (in situ or ex situ), the accuracy of the results (qualitative or quantitative), and the dimension of detection (2D or 3D). Here, the methods are discussed based on their detection dimensions.

### 4.1. 2D Analysis

Normally, CLSM, SEM, and CRM could probe the 2D structural image of the gluten network, although they provide different results (qualitative or quantitative) and are operated in different modes (in situ or ex situ).

#### 4.1.1. CLSM

**Figure 5:**
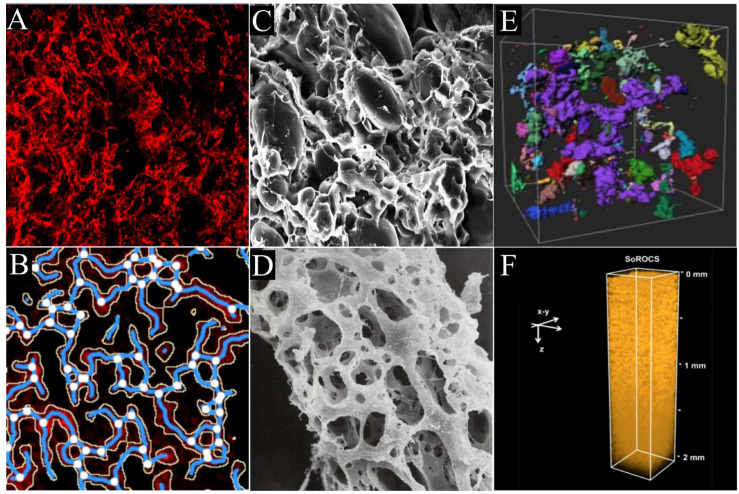
Microscopic images of gluten network in dough by confocal laser scanning microscopy (**A**,**B**), scanning electron microscope (**C**,**D**), and two-photon excitation microscopy (**E**,**F**). (**A**) Dough stained with Rhodamine B. (**B**) The resulting image of A processed with AngioTool64 (white = junctions; blue = gluten-network skeleton; yellow = gluten-thread outline/area). (**C**) Original image. (**D**) Dough after enzymatic removal of starch (adapted with permission from Ref. [100], licensed under CC BY 4.0.). (**E**) 3D structure of gluten (adapted from Ref. [99], licensed under CC BY 4.0). (**F**) 3D reconstruction of gluten treated with SoROCS (adapted with permission from Ref. [99], licensed under CC BY 4.0.).

CLSM is one of the most accurate in situ methods for visualizing gluten microstructures. It reconstructs the 2D image of gluten networks via fluorescence labeling technology, in which a fluorescent dye, commonly Rhodamine B (excitation at 543 nm and emission at 590 nm), is used to stain the protein component in dough samples [85]. Although Rhodamine B is the most common choice, other fluorochromes such as fluorescein isothiocyanate (FITC) and acid fuchsin are also applicable [95,96]. These dyes function via distinct mechanisms: FITC forms covalent bonds with primary amines, acid fuchsin binds electrostatically to cationic groups, whereas Rhodamine B interacts non-covalently with the protein phase via hydrophobic forces. Despite these differences, Rhodamine B is often preferred for its efficient diffusion in bulk-stained dough. In this technique, Rhodamine B can be introduced in two ways: dissolving it in bulk water for kneading and dropping it onto dough slices. Here, they are defined as bulk-water and drop-wise staining methods, respectively (Supplementary File S15). CLSM belongs to the in situ and quantitative methods, which directly work with dough slices and can provide a series of structural parameters of the gluten network, including protein area, protein percentage area, protein junctions, junction density, total/average protein length, protein end-points, end-point rate, lacunarity, branching rate, and protein width (Figure 5). Normally, these parameters are obtained by analyzing the visualized CLSM images using the software Angio Tool64 Ver. 0.6a, called protein network analysis [97]. In contrast to the drop-wise staining method, the bulk-water method consumes much more Rhodamine B. Conversely, the drawbacks of the drop-wise staining method include the limited diffusion depth of Rhodamine B (normally the top few micrometers) and the disruption to the gluten network by the swelling of dough components due to excess water. The possible disruptions include an increase in end-point rate and decreases in average protein length and branching rate [98]. More interestingly, it has been reported that, with the bulk-water method, the dough slices can be prepared at ambient temperature, omitting the freezing program [99]. Thus, in this case, the bulk-water method is simpler than the drop-wise method in operation and free from the damage of the gluten network by freezing, such as a decrease in branching rate and an increase in end-point rate. Due to health risks and the cost of the dye, the bulk-water method is not suitable for the following scenarios: (1) to simultaneously conduct both the network structure characterization and sensory evaluation on the same sample, and (2) to monitor the dough samples in industrial productions.

#### 4.1.2. SEM

By scanning the samples with a highly focused electron beam, SEM is a very useful tool in characterizing the surface ultrastructure of particles or the cross-section of an object with a resolution up to 1 nm [22]. Due to the poor penetrating ability of the electron beam, the samples are always tested in a high vacuum atmosphere to ensure test effectiveness. Up to now, SEM can work with either dough pieces (in situ method) or isolated gluten networks (ex situ method), but both are only capable of describing the gluten network qualitatively until now (Figure 5). It must be noted that the samples of SEM must be thoroughly dried; otherwise, they will undergo serious water loss and structural deformations upon the test in a high vacuum chamber (Supplementary File S16). Hydrated samples are either freeze-dried or critical-point-dried (CPD) after glutaraldehyde fixation. When in situ and ex situ methods are compared, the former is rather simpler and more favorable to probe gluten network structure without structural damages [41], while the latter always results in much clearer images due to the absence of interferences from other dough components [100]. Thus, the in situ method is highly recommended to explore the submicroscopic structures of the gluten network, such as consistency, from a broader perspective, while the ex situ method is particularly suitable to probe fine or microscopic structures, such as lamellar and porous structures, from a narrower perspective. In the ex situ method, water-soluble and water-dispersible components, such as pentosans, starch, and non-gluten proteins, are removed from dough to result in an isolated gluten network, which can be achieved in two ways, namely, washing the dough pieces (washing method) with a solution and hydrolyzing it by amylolytic enzymes (enzymatic method). Commonly, a solution of 0.2 M NaCl and *α*-amylase is used in washing and enzymatic methods, respectively. By contrast, the washing method is more widely used but brings about more structural perturbations to the gluten network, whereas the enzymatic method often suffers from incomplete removal of starch granules [101,102]. For ex situ methods, it is assumed that a quantitative result could be obtained by analyzing the resulting images with the software of ImageJ 1.54p [103], but, unfortunately, this is not evidenced in references up to now.

#### 4.1.3. CRM

CRM combines the chemical sensitivity of Raman spectroscopy with the spatial resolution advantages of confocal microscopy [104]. Thus, with a laser excitation, the spatial distribution of different components of a dough sample is recorded by CRM in terms of a chemical fingerprint image [56]. From the resulting image, a precise localization of the gluten network on a focal plane in the dough can be obtained as a monochrome image defined by the intensity of amide peak I (1600~1700 cm^−1^) (Supplementary File S17). In principle, CRM is similar to CLSM to a great extent but works without fluorescent dye. Both of them belong to in situ methods and illustrate the spatial distribution of dough components in color images [105]. However, they differ in the following aspects: (1) Unlike the quantitative ability of CLSM, CRM generally gives a qualitative description of gluten work, mainly because it has lower resolution than CLSM; (2) besides their common capacities in illustrating the structure of the gluten network, CRM is superior to CLSM due to its extra capacity in reporting the conformational insights of gluten proteins, such as S-S confirmation, the composition of secondary structures, and the states of tertiary and quaternary structures (*I*_830_/*I*_850_) [106]; and (3) CRM offers slower acquisition, lower spatial resolution, and more complex data processing than CLSM. Thus, in characterizing the fine structure of the gluten network, CLSM is the mainstream choice due to its fast, high-contrast, standardized imaging.

### 4.2. 3D Analysis

Definitely, 2D images are inferior to 3D images in displaying the overall and entire realistic structures of an object. With 3D images, objects can be observed from different perspectives, providing more accurate and realistic structural information. Notably, in contrast to 2D images, the capture of 3D images requires more complex and higher-cost techniques. Nowadays, it is still a rather challenging task to obtain the 3D structure of the gluten network in dough, though SEM and CRM are reported to be capable of giving some 3D insights. It is of epoch-making significance that a 3D structure of a gluten network at a millimeter-scale depth (~2 mm) and a submicron resolution (~0.54 μm) is successfully recorded by Ogawa and Matsumura [99], in which an optical clearing reagent (a salicylate-based reagent that optically clears, starchy-products, SoROCS) is combined with two-photon microscopy (2PEM) or CLSM (Figure 5). In more detail, SoROCS is designed to adjust the refractive indices of dough components, eliminate the refractive-index mismatches among them, and thus make the dough specimen transparent. It is found that the specimen undergoes an obvious volume expansion upon the treatment with SoROCS, but this does not significantly impact the skeletal structures of the gluten network. To facilitate the subsequent imaging with 2PEM or CLSM, a thiol-specific fluorescence label of Thiolite^TM^ Green or Alexa Fluor^TM^ 633 C5 Maleimide is used in SoROCS to stain the gluten network. By analyzing the resulting images with Angio Tool, the same quantitative parameters of the gluten network could be concluded as with CLSM in 2D. It is rather exciting that no significant differences were observed in these parameters between the present method and the in situ CLSM 2D method as mentioned above. Thus, this method can be assumed to be another in situ and quantitative technique capable of probing the structure or gluten network (Supplementary File S18).

In comparison, the present method can conduct quantitative analysis of the gluten network as does CLSM in 2D, but it is much more complicated in operation and time-consuming, especially the optical clearing of the specimen. Definitely, the present method is superior to the 2D CLSM due to its ability to view the images of desired regions and planes in any orientation, thus resulting in a better understanding of the gluten structure. For the present method, two setups, SoROCS-2PEM and SoROCS-CLSM, can be applied, but they differ from each other in the following two aspects: (1) 2PEM offers a higher resolution (~0.54 μm) as well as a higher imaging depth (~2 mm) than CLSM (~30 mm and ~600 μm); and (2) the apparatus of CLSM is much cheaper and more easily available than 2PEM.

A comprehensive comparison of various characterization methods reveals that CLSM is the preferred technique for in situ quantitative characterization of gluten networks, providing multiple structural parameters, though it necessitates fluorescent staining [85]. SEM is ideal for high-resolution observation of micro- and ultra-micro surface morphologies; however, as samples are prone to deformation during drying, it is primarily used for qualitative analysis [22]. CRM can simultaneously acquire spatial distribution and chemical structural information without fluorescent labeling, but its imaging speed is relatively slow and largely qualitative [56]. 3D techniques based on optical clearing can precisely restore the 3D network structure and serve as powerful tools for studying spatial conformations; yet they are constrained by complex pretreatment and high costs [99]. In practice, the choice of technology should align with specific research objectives: CLSM is prioritized for routine structural quantification; SEM is selected for micromorphological observation; CRM is employed for label-free chemical imaging and structural analysis; and 3D optical imaging is reserved for spatial structure and mechanistic studies.

Overall, despite the capability of 3D characterization techniques to provide a more authentic and comprehensive restoration of the spatial structure and connectivity of gluten networks, 2D analysis methods remain widely utilized in gluten structure research. The core reasons for this prevalence lie in the inherent advantages of 2D methods, including simple sample preparation, rapid detection, low cost, mature quantitative algorithms, and suitability for high-throughput sample screening. In contrast, while 3D characterization excels in structural display, it also suffers from cumbersome operation, long processing time, and high equipment cost, making it unsuitable for rapid routine sample characterization. Therefore, 2D methods are optimal for routine structural quantification and rapid screening, while 3D methods are better suited for in-depth analysis of spatial conformations and formation mechanisms.

## 5. Challenges and Perspectives

Despite the aforementioned great advances in elucidating the multi-scale structures of gluten proteins in dough by the combination of various modern analytical techniques, such as electrophoresis, chromatography, mass spectrometry, microscopic imaging, and proteomics, there are many challenges that persist in this field due to the incredible complexity of gluten proteins, especially the structure of the gluten network. For the present techniques, the following defects should be addressed in future investigations.

(1) In the fractionation of gluten proteins, the solubility-based techniques often encounter a non-negligible cross-contamination, which leads to inaccurate, even erroneous, results for the quantification and component identification of gluten fractions.
(2) In characterizing the tertiary and quaternary structures of gluten proteins, the present techniques can only obtain indirect, local, and rather rough information, which is not at all sufficient for a comprehensive and complete understanding of intended structures.
(3) As for the characterization of gluten aggregates, the applied separation techniques are almost impossible not to disrupt the native structures of gluten aggregates in dough, which certainly leads to false or dishonest results.
(4) In capturing the 2D and 3D images of the gluten network, the current techniques have a limited field of view, both in the sectional area and depth, which may neglect the spatial differences of the gluten network in dough and put the results at risk of taking part for the whole.
(5) The present techniques, even specific techniques, demonstrate substantial differences in testing procedures and conditions among different literature, which makes their results incomparable. Thus, the standardization of present techniques is of high importance in generalizing the test results.
(6) Theoretically, there should be a hierarchical relationship between structural scales of gluten proteins, where molecular structures determine aggregate structures, and aggregate structures cross-link into network structures. In this view, although the present techniques provide information for different scales, they are not always interpreted in an integrated manner, and the assumed hierarchical relationship is not well established.
(7) Actually, the multi-scale structures of gluten proteins are not static events, but they undergo continuous dynamic changes during the formation and processing of the dough, and the results are often sample-specific. Regrettably, the fundamental laws of these changes at different scales as well as their evolution, need further clarification.

## 6. Conclusions

The multi-scale structures of gluten proteins at molecular, aggregate, and network sizes are primary determinants of dough properties and the quality of flour-based foods, thus deserving fine characterization. Honestly, the multi-scale structures of gluten proteins are some of the most complex structures in the food system. Hence, a series of useful and informative techniques should be adopted for their characterization. In recent years, great advances have been achieved for techniques to illustrate the structure of gluten proteins at different scales, such as electrophoresis, RP-HPLC, proteomics, FT-IR, and FT-Raman at the molecular scale; SEC, AsFlFFF, DLS, MALS, and DRI/UV at the aggregate scale; and CLSM, SEM, CRM, and 2PEM at the network scale, respectively. In reviewing these methods, the sister techniques for the same structural parameter are compared with an aim to provide scenario-directed guidance for technique selection. Moreover, a typical example for each technique is recommended and scheduled in principle, apparatus, reagents, operation diagram, and result presentation, which will facilitate the utilization of these techniques, especially for new researchers in this field. Finally, the challenges and perspectives on this topic are discussed to provide focusing points in improving these techniques or developing novel techniques for this task.

## Data Availability

No new data were created or analyzed in this study. Data sharing is not applicable to this article.

## Supplementary Materials

The following supporting information can be downloaded at: https://www.mdpi.com/article/10.3390/biom16030382/s1, File S1: Fractionation of gluten fractions—Osborne/Sequential extraction method; File S2: Analysis of monomeric/subunit composition of gluten fractions—sodium dodecyl sulfate polyacrylamide gel electrophoresis; File S3: Analysis of monomeric/subunit composition of gluten fractions—2-dimensional gel electrophoresis; File S4: Analysis of monomeric/subunit composition of gluten fractions—size-exclusion high performance liquid chromatography; File S5: Analysis of monomeric/subunit composition of gluten fractions—reversed-phase high-performance liquid chromatography; File S6: Analysis of monomeric/subunit composition of gluten fractions—Proteomics; File S7: Structure analysis of gluten proteins—Fourier transform infrared spectroscopy; File S8: Structure analysis of gluten proteins—Fourier transform Raman spectroscopy; File S9: Analysis of the covalent bonds—spectrophotometric method; File S10: Structure analysis of gluten proteins—fluorescence spectroscopy; File S11: The degree of overall aggregation—front-face fluorescence method with the probe of thioflavin T; File S12: The degree of covalent and non-covalent aggregations—the solubility method; File S13: Analysis of the morphology (size and shape) of gluten aggregates; File S14: Analysis of the content of non-covalent bonds involved in gluten aggregates —solubility method; File S15: Analysis of the network structure of gluten proteins—confocal laser scanning microscopy; File S16: Analysis of the network structure of gluten proteins—scanning electron microscopy; File S17: Analysis of the network structure of gluten proteins—confocal Raman microscopy; File S18: Analysis of the network structure of gluten proteins—two-photon excitation microscopy.

## Abbreviations

The following abbreviations are used in this manuscript:

GMP	Glutenin macropolymer
HMW-GS	High-molecular-weight glutenin subunits
LMW-GS	Low-molecular-weight glutenin subunits
SDS-PAGE	Sodium dodecyl sulfate polyacrylamide gel electrophoresis
2-DE	2-dimensional gel electrophoresis
SE-HPLC	Size-exclusion high-performance liquid chromatography
RP-HPLC	Reversed-phase high-performance liquid chromatography
MS	Mass spectrometry
SS	Disulfide bond
SH	Sulfhydryl
FT-IR	Fourier transform infrared spectroscopy
FT-Raman	Fourier transform Raman spectroscopy
*β*-ME	*β*-mercaptoethanol
TCEP	Tris (2-carboxyethyl) phosphine
Tyr	Tyrosine
Trp	Tryptophan
Phe	Phenylalanine
SEC	Size-exclusion chromatography
AsFlFFF	Asymmetrical flow field-flow fractionation
UV	Ultraviolet absorbance
DRI	Differential refractive index
MALS	Multi-angle light scattering
DLS	Dynamic light scattering
PDI	Polydispersity index
CLSM	Confocal laser scanning microscopy
CRM	Confocal Raman microscopy
SEM	Scanning electron microscopy
2PEM	Two-photon excitation microscopy
SoROCS	Salicylate-based reagent optically clears starchy products
LC-MS/MS	Liquid chromatography-tandem mass spectrometry
NanoLC-Orbitrap-MS/MS	Nanoflow liquid chromatography-Orbitrap tandem mass spectrometry
NanoLC-ESI-QqTOF-MS/MS	Nanoflow liquid chromatography-electrospray ionization-quadrupole-quadrupole-time-of-flight tandem mass spectrometry
