# Supplementary material for: Key Methodologies in Characterizing the Multi-Scale Structures of Gluten Proteins in Dough: A Comparative Review
Source: Biomolecules. 2026 Mar 3;16(3):382. doi: 10.3390/biom16030382 (PMC13023611; doi:10.3390/biom16030382)
Supplement: Supplementary file 1 [file biomolecules-16-00382-s001.zip › Supplementary File S11.pdf]

Supplementary material S11:

The degree of overall aggregation—front-face fluorescence method with the probe of thioflavin T

Principle

During gluten aggregation, protein molecules form fibrillar β-sheet assemblies with a characteristic cross-*β* conformation. Thioflavin T (ThT) selectively intercalates along the groove sites of these fibrillar β-sheets, restricting its intramolecular rotation and causing a marked enhancement and spectral shift of fluorescence. These spectral changes are negligible for monomers/subunits, small oligomers, or aggregates lacking cross-*β* structure. Using front-face fluorescence (FFF) geometry to study the extent of fibrillar *β*-sheet formation, and thus the overall aggregation degree of gluten proteins, is quantified by the maximum fluorescence intensity at 482 nm upon excitation at 435 nm.

Apparatus

1. Fluorescence spectrometer, equipped with an optical fiber: used to measure the fluorescence intensity of dough samples by inserting the optical fiber into the samples, and to collect thioflavin T emission spectra (350-600 nm with excitation at 435 nm).

Reagents

1. Thioflavin T solution (50 μM): used to replace water for mixing with wheat flour, for analyzing dough protein aggregation.

Procedure

1. Preparation of the sample

Dough is prepared by mixing 500 g of wheat flour (Nisshin Seifun, crude protein 8.5%, ash 0.34%) with 160 g of 50 μM thioflavin T, followed by kneading using a mixer (Hobart, N50) for 20 min at 139 rpm to produce a wheat dough.

The dough samples are collected at different time points during the mixing process.

Note: the water is replaced with 50-μM thioflavin T to analyze the dough protein aggregation.

2. Collect fluorescence spectra

Immediately after sampling, the dough is covered with plastic wrap, and an optical fiber from the fluorescence spectrometer is pushed into the samples to measure the fluorescence intensity.

Next, thioflavin T fluorescence is monitored by the emission spectra from 350 to 600 nm, with excitation at 435 nm, and the resultant spectra were subsequently normalized to the maximum.

The normalized maximum value in the emission spectra is assumed to correspond to the fluorescence intensity, which was then compared between samples.

3. Workflow diagram

An overview of the workflow used to assess the degree of overall aggregation shown in Fig. 1.

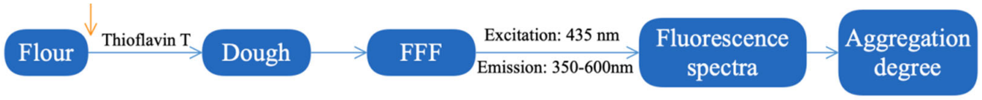

Fig. 1. Workflow of FFF for assessing the degree of overall aggregation.

References

Iwaki, S., Hayakawa, K., Fu, B.-X., & Otobe, C. (2021). Changes in Hydrophobic Interactions among Gluten Proteins during Dough Formation. *Processes*, *9*, 1244. https://doi.org/10.3390/pr9071244

Sadat, A., Corradini, M. G., & Joye, I. J. (2022). Vibrational and fluorescence spectroscopy to study gluten and zein interactions in complex dough systems. *Food Science*, *5*, 479-490. https://doi.org/10.1016/j.crfs.2022.02.009
