# Supplementary material for: Key Methodologies in Characterizing the Multi-Scale Structures of Gluten Proteins in Dough: A Comparative Review
Source: Biomolecules. 2026 Mar 3;16(3):382. doi: 10.3390/biom16030382 (PMC13023611; doi:10.3390/biom16030382)
Supplement: Supplementary file 1 [file biomolecules-16-00382-s001.zip › Supplementary File S12.pdf]

Supplementary material S12：

The degree of covalent and non-covalent aggregations—the solubility method

Principle

The solubility method distinguishes covalent and non-covalent aggregation of gluten proteins based on their differential solubility in SDS solutions. Non-covalent interactions (hydrophobic interactions, hydrogen bonds, and electrostatic forces) are readily disrupted by SDS, and the solubilized fraction therefore represents the non-covalent component. By applying SDS at increasing concentrations (0.1%, 0.3%, and 0.5%), the non-covalent component can be further subdivided into weak, medium, and strong fractions, corresponding to bonds of different strength. In contrast, covalent aggregation, primarily stabilized by disulfide bonds, remains insoluble even in 0.5% SDS, and the SDS-insoluble fraction is thus regarded as the covalent component.

Reagents

1. Phosphate buffer solution (PBS, 50 mM, pH 7.0): used as an extraction buffer for gluten proteins.

2. Sodium dodecyl sulfate (SDS) buffers: prepared in PBS at concentrations of 0.0%, 0.1%, 0.3%, and 0.5% (w/v); used as a detergent to disrupt non-covalent protein interactions.

Procedure

1. Preparation of the sample

Dough is prepared by mixing 500 g of wheat flour (Nisshin Seifun, crude protein 8.5%, ash 0.34%) with 160 g of deionized water, followed by kneading using a mixer for 20 min at 139 rpm to produce a wheat dough.

2. Total protein (P_t) determination

Fresh dough (1 g) is analyzed for total protein content (P_t) using the Kjeldahl method.

3. Sequential Extraction with SDS Buffers

Fresh dough (1 g) is homogenized with 20 mL of 50 mM phosphate buffer (pH 7.0) containing SDS at 0.0%, 0.1%, 0.3%, or 0.5% (w/v) at 10,000 rpm for 5 min using a homogenizer.

For the 0.5% SDS treatment, the suspension is further subjected to ultrasonic extraction for 15 s at 60% power (76 W) to facilitate protein solubilization without disrupting covalent bonds.

The resulting suspensions are centrifuged at 5000×*g* for 10 min, and the supernatants are carefully collected.

Residual pellet is re-extracted with the corresponding buffer, followed by centrifugation under the same conditions.

The two supernatants obtained from each extraction step were combined, and the protein content was determined by the Kjeldahl method. The extracted protein fractions are designated as P_0 (0.0% SDS), P_1 (0.1% SDS), P_2 (0.3% SDS), and P_3 (0.5% SDS), respectively.

The degrees of non-covalent aggregation (with contributions from weak, medium, and strong interactions) and covalent aggregation were quantified according to equations (1)-(5), based on the sequential SDS extraction method.

4. Workflow diagram

An overview of the analysis the degree of covalent and non-covalent aggregations workflow is shown in Fig. 1.

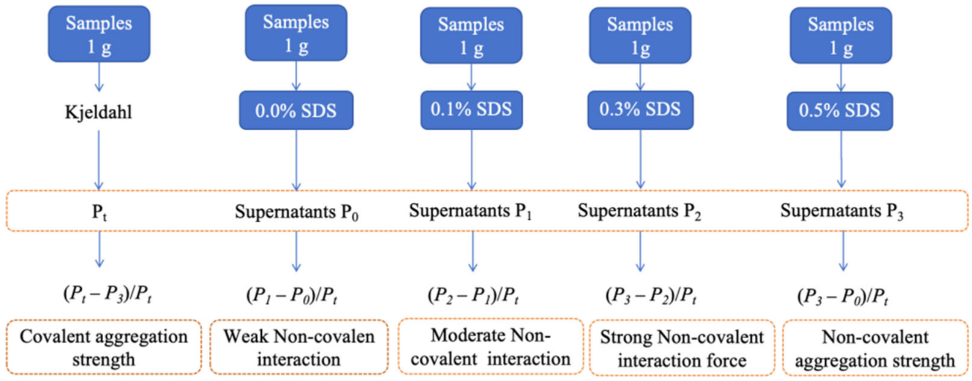

Fig. 1. Workflow for analyzing the degree of covalent and non-covalent aggregations.

Result presentation

Calculation of aggregation degree:

Non-covalent aggregation (%) $=(P_3-P_0)/P_t\times100\%$ (1)

Contributions of different interaction strengths:

Weak$=(P_1-P_0)/P_t\times100\%$ (2)

Medium$=(P_2-P_1)/P_t\times100\%$ (3)

Strong $=(P_3-P_2)/P_t\times100\%$ (4)

Covalent aggregation:

Covalent aggregation (%) $=(P_t-P_3)/P_t\times100\%$ (5)

Where, P_t denotes the total protein content (mg). P_0, P_1, P_2, and P_3 represent protein content (mg) extracted with 0.0%, 0.1%, 0.3%, and 0.5% SDS, corresponding to baseline soluble proteins, weak, medium, and strong non-covalent interactions, respectively. The fraction (P_t-P_3) indicates SDS-insoluble proteins, mainly stabilized by covalent cross-links.

References

Iwaki, S., Aono, S., Hayakawa, K., Fu, B. X., & Otobe, C. (2020). Changes in Protein Non-Covalent Bonds and Aggregate Size during Dough Formation. *Foods*, *9*, 1643. https://doi.org/10.3390/foods9111643
