# Supplementary material for: Key Methodologies in Characterizing the Multi-Scale Structures of Gluten Proteins in Dough: A Comparative Review
Source: Biomolecules. 2026 Mar 3;16(3):382. doi: 10.3390/biom16030382 (PMC13023611; doi:10.3390/biom16030382)
Supplement: Supplementary file 1 [file biomolecules-16-00382-s001.zip › Supplementary File S13.pdf]

Supplementary material S13：

Analysis of the morphology (size and shape) of gluten aggregates

Principle

Accurate characterization of protein aggregate size requires a comprehensive analytical workflow that encompasses controlled extraction, precise separation, and multi-parameter quantification. Specifically, appropriate chemical reagents and treatment methods should be selected according to the research objectives to enable the extraction of gluten protein aggregates. The obtained fractions are subsequently separated by size-based fractionation techniques such as size-exclusion chromatography (SEC) or asymmetrical flow field-flow fractionation (AsFlFFF). Finally, to characterize the size-different components of gluten aggregates, a profile of consecutive eluates from SEC or AsFlFFF is recorded against elution time. By analyzing this profile, the mass, size, molecular weight, and shape of each component can be concluded. According to the purpose of the test, the profile is often recorded by selectively using one or more concentration-sensitive detectors, such as ultraviolet absorbance (UV), differential refractive index (DRI), dynamic light scattering (DLS), and multi-angle light scattering (MALS).

Apparatus

1. High-performance liquid chromatography (HPLC) system (Dionex Ultimate 3000 HPLC system): used with a SEC column to control mobile phase flow rate (0.7 mL/min) for chromatographic separation of protein components, and with an asymmetrical flow field-flow fractionation system to regulate fluid flow in the AF4 channel (controlling sample injection, focusing, and elution).

2. SEC column. Analytical column: TSK G4000 SWXL (30×7.8 cm), guard column: TSK 3000-SW (4×6 cm). Used as an SEC stationary phase to separate proteins by molecular size, and the guard column prevents impurity contamination of the analytical column to extend its service life.

3. Asymmetrical flow field-flow fractionation (AsFlFFF) system: with channel dimensions of L = 26.5 cm, b_0 = 2.1 cm, b_L = 0.6 cm, spacer thickness of 350 μm; using WE as mobile phase to achieve size-based fractionation of gluten proteins (monomers, polymers, assemblies) via focusing (1.5 mL/min, 6 min) and gradient elution (1.5 → 0.10 mL/min, 22 min).

4. Multi-detector system: UV detector, DRI detector, MALS detector, and DLS detector. It can simultaneously obtain key parameters.

5. Data processing software (Wyatt Technology Corp, ASTRA 6.1.6) uses the Berry extrapolation method to process multi-detector data and calculate key indicators of gluten proteins.

Reagents

1. NaCl solution (2%, w/v): prepared by accurately weighing 2.0 g of analytical-grade NaCl, dissolving it in 100 mL of deionized water, and stirring until fully dissolved; used to repeatedly wash fresh dough to remove starch.

2. Ethanol-water mixed solvent (WE, 50/50 v/v): used for mild solubilization of gluten proteins during extraction and as the mobile phase for AsFlFFF.

3. Filter membrane (0.45 μm); used to filter SEC samples to remove micro-impurities and avoid SEC column clogging.

4. Denaturing buffer (0.1 M sodium phosphate, 1% w/v sodium dodecyl sulfate (SDS), 6 M urea, pH 6.8): used for SEC sample pretreatment to disrupt non-covalent bonds between gluten proteins.

5. Sodium phosphate buffer (0.1 M, PBS; 0.1% w/v SDS): used as the SEC mobile phase to elute protein components in the column and maintain protein solubility during separation.

6. Filter membrane (1.2 μm): used to filter AsFlFFF samples to remove large particles and aggregates, protecting the AsFlFFF channel and membrane.

7. Regenerated cellulose membrane (10 kDa molar mass cutoff, compatible with AsFlFFF Eclipse channel 350 μm spacer): installed at the bottom of the AsFlFFF channel as the accumulation wall to retain small-molecule components during AsFlFFF separation and control protein retention/elution for size-based fractionation.

Procedure

1. Samples

Dough is prepared by mixing 500 g of wheat flour (Nisshin Seifun, crude protein 8.5%, ash 0.34%) with 160 g of deionized water, followed by kneading using a mixer for 20 min at 139 rpm to produce a wheat dough.

Take fresh dough and wash it repeatedly with NaCl solution until the filtrate is clear and there is no blue reaction with iodine solution. This process is continued until the water used to rinse the dough no longer turns blue when tested with iodine solution, yielding wet gluten. The isolated wet gluten is freeze-dried, then ground and passed through an 80-mesh sieve to obtain gluten protein powder.

2. Extraction

A "mild" protein extraction is performed in a mixture of WE solution. Gluten (20 g) is added to 200 mL of the WE solvent and mixed at 60 rpm for 19 h at 20 °C. After centrifugation (11,000 rpm, 20°C, 30 min), the supernatant is cooled to 6 °C for 12 h to induce liquid-liquid phase separation. The dense phase is isolated after centrifugation (11,000 rpm, 6 °C, 30min). Five volumes of deionized water are added per volume of dense phase. The mixture is then frozen at -40 °C and subsequently freeze-dried and powdered.

3. Fractionation

3.1 SEC

SEC analysis of gluten proteins is carried out on a Dionex Ultimate 3000 HPLC system equipped with a TSK G4000 SWXL column preceded by a TSK 3000-SW guard column (4×6 cm).

Samples are prepared at a protein concentration of C ¼ 4 g/L in WE solvent, filtered through a 0.45 μm filter, and then diluted to reach a concentration of about 1 g/L in a denaturing buffer. Elution of the injected sample (20 μL) is performed at 0.7 ml/min in sodium phosphate buffer.

3.2 AsFlFFF

Directly disperse the freeze-dried gluten powder in the WE solvent, prepare it to a concentration of 2 mg/mL, and gently stir it at room temperature for 2 h before it is ready for sample injection.

The protein solutions are filtered through a 1.2 μm filter, then 20 μL aliquots are separated using an AsFlFFF system combined with a Dionex Ultimate 3000 HPLC system that regulates the flow into the channel during the fractionation. The separation occurs in a long, asymmetrical channel.

Sample injection starts at time 3 min for a duration of 3 min with a flow rate of 0.2 mL/min. The focus step starts at 2 min and lasts 6 min with a crossflow rate of 1.5 mL/min. At the end of the focus step, the elution step starts with a crossflow rate fixed at 1.5 mL/min for 20 min, allowing the separation of the smallest objects. To facilitate the elution of the largest objects, a linear decrease of the crossflow from 1.5 to 0.10 mL/min in 22 min is imposed. Then, the crossflow is maintained at 0.10 mL/min for 10 min. Once the crossflow is stopped, the injection loop is held for 5 min. The detector outlet flow is fixed at 0.6 mL/min to accommodate the high viscosity of the WE solvent (η_0 = 2.455 mPa s at 25 °C).

3.3 Batch DLS measurements

Batch mode DLS measurements are performed at 25 °C using a Zetasizer Nano ZS equipped with a 633 nm laser and operating in backscatter detection. Prior to measurements, all samples are diluted in PBS to 1 mg/mL (total lipid concentration). The size results were obtained by averaging 5 consecutive measurements. The results of the cumulant analysis, size scale in hydrodynamic radius (R_h) and polydispersity index (*PDI*), are reported. Intensity-weighted hydrodynamic size distributions

generated by nonnegative constrained least squares analysis are also reported where appropriate.

3.4 Online DLS measurements

Online DLS measurements performed using either a Wyatt QELS (Quasi-Elastic Light Scattering) integrated directly with the MALS at an angle of 99.9° or 134° or using a Malvern Zetasizer positioned as the last detector and operating in flow-mode with backscatter detection at 173°. The measured correlation functions obtained during fractionation analyzed using either single exponential decay or the cumulants method, both of which should theoretically yield the same value at the same angle if the eluting sample is size-fractionated (i.e., monodisperse). The output is the equivalent sphere hydrodynamic radius.

3.5 Online MALS measurements

The online MALS detectors are calibrated at a scattering angle of 90°, and the remaining detector angles are normalized to the response at 90° using an isotropic scatterer (e.g., BSA in PBS) according to manufacturer recommendations. This process yields absolute scattering intensity (Rayleigh ratio, cm^{-1}) and ensures that all detector angles perform equally. The excess Rayleigh ratio is determined for fractionated samples (i.e., scattering from the pure mobile phase is subtracted from the sample signal). The resulting absolute intensity at 90° is typically presented in a self-normalized manner. For each "slice" or data point in a fractogram, the excess Rayleigh ratio is analyzed versus scattering angle and fit with an appropriate scattering equation (e.g., Berry form of the Debye model in the case of the sphere form factor in the case of PSL). The number of angles and angular range vary for different detectors. Data points are selected based on the quality of the fit, and the output is the root mean square radius (commonly referred to as the radius of gyration), R_g, where $R_g = \sqrt{3/5}R_s$ and R_s is the geometric radius of a solid sphere. Furthermore, the second virial coefficient (A_2) has also been reported.

Best Practice Note: Calibration and normalization should be performed at least once

per year and anytime the MALS flow cell has been disassembled (e.g., for cleaning). Normalization of all detectors to 90° should be confirmed, and, if necessary, corrected each time the mobile phase composition is changed.

3.6 Online UV absorbance

Online UV-Vis absorbance at 280 nm is utilized for mass detection during fractionation and for the determination of the analyte mass recovery.

3.7 Online DRI measurements

Confirm that the flow path between AsFlFFF and DRI is correctly connected: The outlet of the AsFlFFF separation channel should be connected by tubing to the DRI detector cell, ensuring the tubing is sealed with no leaks to prevent air bubbles. Set the response time based on the separation speed to avoid peak broadening. Inject the sample into the FFF system, where it will be separated by size before entering the DRI detector cell in order of size. Simultaneously record FFF separation parameters (such as field strength changes and time) and the signal curve from the DRI. Use workstation software (e.g., Omnisec, Clarity) to link both data sets and obtain a 'retention time-refractive index signal' profile to analyze the sample's size distribution and concentration fractions.

3.8 Data processing

In addition to the individual parameters obtained above, R_g and R_h are reported on the fractogram across the full width at half maximum (FWHM) for fractionated peaks that are adequately defined, or near the peak or shoulder maximum if ill-defined. In tabulated results, R_g and R_h are averaged across the FWHM (for monomodal samples) or reported at peak maxima (for polymodal samples). The spread of size values across the FWHM is reported as a measure of peak polydispersity (where spread = difference between the minimum and maximum size values across the FWHM). To perform these measurements and to present data in a fractogram format, scattering intensity at 90° is used for R_g and R_h (measured by QELS in the MALS flow cell), whereas for R_h measured using a Zetasizer in flow mode, the detector count rate (unattenuated) at the

angle of measurement (173°) is used instead. Finally, the Burchard-Stockmayer shape factor is calculated from the ratio of the root mean square radius and the hydrodynamic radius ($\rho = R_g/R_h$).

Moreover, the detector combination of MALS-UV/DRI can measure number average molecular weight (M_n) and weight average molecular weight (M_w), thus further narrating the molecular weight distribution of aggregates in terms of polydispersity index ($PDI=M_n/M_w$).

3.9 Workflow diagram

An overview of the analysis of the appearance of gluten aggregates workflow is shown in Fig. 1.

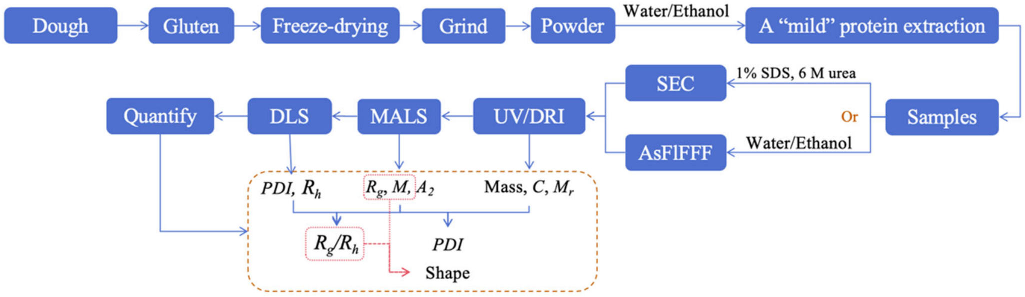

Fig. 1. Workflow for analyzing the appearance of gluten aggregates.

Result presentation

SEC/AsFlFFF-DLS-MALS-DRI/UV setups possess the most comprehensive functions. Notably, these detectors have different detection capabilities. Among them, UV and DRI are the most basic ones, which conclude the following parameters: the concentration of size-fractioned components as well as their mass and relative molecular weight (M_r). DLS can provide size scale in terms of hydrodynamic radius (R_h) and polydispersity index (*PDI,* based on hydrodynamic radius), while MALS can give absolute molecular weight (M), size scale in rotation radius (R_g), and particle interaction intensity in second virial coefficient (A_2). In addition to these, the shape of gluten aggregates can also be characterized by jointly using DLS and MALS, because the derivative parameter of the R_g/R_h ratio is aggregate shape dependent. Moreover, the

detector combination of MALS-UV/DRI can measure number average molecular weight (M_n) and weight average molecular weight (M_w), thus further narrating the molecular weight distribution of aggregates in terms of polydispersity index ($PDI= M_n/M_w$).

References

Morel, M.-H., Pincemaille, J., Chauveau, E., Louhichi, A., Violleau, F., Menut, P., Ramos, L., & Banc, A. (2020). Insight into gluten structure in a mild chaotropic solvent by asymmetrical flow field-flow fractionation (AsFlFFF) and evidence of non-covalent assemblies between glutenin and ω-gliadin. *Food Hydrocolloids*, *103*, 105676. https://doi.org/10.1016/j.foodhyd.2020.105676

Iwaki, S., Fu, B. X., & Hayakawa, K. (2023). Behavior of protein aggregates via electrostatic interactions or hydrogen bonds during dough formation. *Journal of Cereal Science*, 111, 103683. https://doi.org/10.1016/j.jcs.2023.103683

Parot, J., Caputo, F., Mehn, D., Hackley, V. A., & Calzolai, L. (2020). Physical characterization of liposomal drug formulations using multi-detector asymmetrical-flow field flow fractionation. *Journal of Controlled Release*, *320*, 495–510. https://doi.org/10.1016/j.jconrel.2020.01.049
