# Supplementary material for: Key Methodologies in Characterizing the Multi-Scale Structures of Gluten Proteins in Dough: A Comparative Review
Source: Biomolecules. 2026 Mar 3;16(3):382. doi: 10.3390/biom16030382 (PMC13023611; doi:10.3390/biom16030382)
Supplement: Supplementary file 1 [file biomolecules-16-00382-s001.zip › Supplementary File S14.pdf]

Supplementary material S14:

Analysis of the content of non-covalent bonds involved in gluten aggregates — solubility method

Principle

Treat samples with solvents that selectively dissociate specific non-covalent interactions and record the ensuing increase in protein solubility, whose magnitude reflects that interaction's relative contribution to aggregate stability.

Reagents

1. Phosphate buffer solution (PBS, 50 mM, pH 7.0): used as an extraction buffer for gluten proteins.

2. Four solvent solutions: 0.1 M NaCl (S_1), 0.6 M NaCl (S_2), 0.6 M NaCl combined with 1.5 M urea (S_3), and 0.6 M NaCl along with 8 M urea (S_4)

Procedure

1. Dough preparation

Dough is prepared by mixing 500 g of wheat flour (Nisshin Seifun, crude protein 8.5%, ash 0.34%) with 160 g of deionized water, followed by kneading using a mixer for 20 min at 139 rpm to produce a wheat dough.

2. Protein extraction

Parallel regime: lyophilized dough (100 mg) is combined with the four solvents (10 mL each) and subjected to a 1 h incubation at 25 °C. Following this, the mixture underwent centrifugation (10,000×*g*, 20 min, 4 °C).

Cascade regime: Mix 0.2 g of dough with 10 mL of S_1 solution, then homogenize for 1 h at 4 °C, and centrifuge (10,000×*g*, 20 min, 4 °C). Remove the supernatant, and store at 4 °C. Add 10 mL of S_2 solution to the resulting precipitate, homogenize (1h, 4 °C) and dissolve with sufficient stirring, then centrifuge (10,000×*g*, 20 min, 4 °C),

and remove the supernatant. Add 10 mL of S_3 solution to the resulting precipitate, homogenize (1h, 4 °C), and dissolve with sufficient stirring, then centrifuge (10,000×*g*, 20 min, 4 °C), and remove the supernatant. To the above precipitate, 10 mL of S_4 solutions are added. This is homogenized (1h, 4 °C) and dissolved with sufficient stirring, and the supernatant is collected after centrifuging at 10,000×*g*, 20 min, 4 °C.

3. Quantitative analysis

Parallel regime: the protein content of the supernatant is determined by using the Kjeldahl method. The protein contents of the proteins dissolved by extractants S_1, S_2, S^3, and S_4 are A, B, C, and D, respectively.

Cascade regime: the protein content of the supernatant is determined by using the Kjeldahl method. The protein contents of the proteins dissolved by extractants S_1, S_2, S_3, and S_4 are A, E, F, and G, respectively.

4. Workflow diagram

An overview of the analysis of non-covalent bond interactions involved in gluten aggregate formation is presented in the workflow shown in Fig. 1.

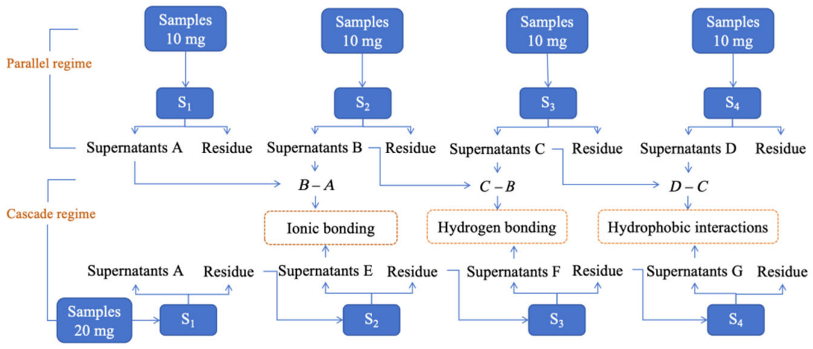

Fig. 1. Workflow for analyzing the content of non-covalent bonds involved in gluten aggregates.

Result presentation

1. Parallel regime

The contribution of ionic bonding, hydrogen bonding, and hydrophobic interactions was expressed as the protein content the stepwise increases in protein content, namely

$B{-}A$, $C{-}B$, and $D{-}C$, respectively.

2. Cascade regime:

The contributions of ionic bonding, hydrogen bonding, and hydrophobic interactions were quantified as the protein content dissolved in the S_2, S_3, and S_4 solutions, denoted as E, F, and G, respectively.

References

Iwaki, S., Fu, B. X., & Hayakawa, K. (2023). Behavior of Protein Aggregates via Electrostatic Interactions or Hydrogen Bonds during Dough Formation. *Journal of Cereal Science*, *111*, 103683. https://doi.org/10.1016/j.jcs.2023.103683

Lin, Q., Shen, H., Ma, S., Zhang, Q., Yu, X., & Jiang, H. (2023). Morphological Distribution and Structure Transition of Gluten Induced by Various Drying Technologies and Its Effects on Chinese Dried Noodle Quality Characteristics. *Food and Bioprocess Technology*, *16*, 1374-1387. https://doi.org/10.1007/s11947-023-02993-7

Liu, H., Liang, Y., Zhang, S., Liu, M., He, B., Wu, X., Yin, H., Zhang, X., & Wang, J. (2024). Physicochemical Properties and Conformational Structures of Pre-Cooked Wheat Gluten during Freeze-Thaw Cycles Affected by Curdlan. *Food Hydrocolloids*, *147*, 109381. https://doi.org/10.1016/j.foodhyd.2023.10938
