# Supplementary material for: Key Methodologies in Characterizing the Multi-Scale Structures of Gluten Proteins in Dough: A Comparative Review
Source: Biomolecules. 2026 Mar 3;16(3):382. doi: 10.3390/biom16030382 (PMC13023611; doi:10.3390/biom16030382)
Supplement: Supplementary file 1 [file biomolecules-16-00382-s001.zip › Supplementary File S15.pdf]

Supplementary material S15:

Analysis of the network structure of gluten proteins—confocal laser scanning microscopy

Principle

Specimens are prepared for analysis with a confocal laser scanning microscope (CLSM) by labeling with the Rhodamine B fluorescent probe. The laser beam, after being spatially filtered through the illumination pinhole, is reflected by the dichroic mirror and focused on the specimen via the objective lens to excite fluorescence. The emitted fluorescence, which has a longer wavelength than the incident light, is allowed to pass through the detection pinhole only when it is in focus at the focal plane, while out-of-focus light is blocked. As a result, only in-focus fluorescence signals are recorded by the detector, enabling point-to-point confocal imaging. The focal plane is scanned in a raster pattern, and the signals are collected by a photomultiplier tube, subsequently processed by the system to generate the fluorescence image. Finally, Image quantitative analysis is performed with protein network analysis by AngioTool64.

Apparatus

1. LSM 800 confocal laser scanning microscopy: obtain the image of the gluten network.

2. CM1950 freezing microtome: dough is sliced (20 μm thickness).

3. Software AngioTool64: used for analyzing CLSM images.

Reagents

1. Rhodamin B solution (0.1 g/L, in water); for solution, dissolve approximately 10 mg solid Rhodamin B in deionized water and dilute to 100 milliliters. The prepared solution should be stored at 4 °C and protected from light to ensure stability.

2. Leica Tissue Freezing Medium: used to fix the samples for subsequent sectioning.

Procedure

1. Staining of dough samples by bulk-water or drop-wise methods.

1.1 Bulk-water method

The bulk water method is performed by replacing 5 mL the bulk water with the Rhodamine B solution and adding it during kneading to the dough. Thus, a homogeneous distribution of dye in the dough was achieved. Dough (2 g) is transferred to an object carrier, cut with a razor blade carefully to achieve a plane surface and sealed afterwards with an object slide. The stained samples are captured for images by CLSM after 10 min of resting time for dough relaxation.

1.2 Drop-wise methods

Dough is sliced (20-μm thickness) in a CM1950 freezing microtome after cryo-embedding with Leica Tissue Freezing Medium. The dough slice is rapidly stained drop-wise with 10 μL Rhodamin B solution (0.1 g/L, in water) at 26 °C for 2 h on object carrier and sealed afterwards with an object slide.

Note: 1) dough can be cut by a razor blade, but if the slices are too thick, the dye may not be able to fully penetrate the sample; 2) during sample staining, rapidly add the staining solution drop-wise to prevent the specimen from drying and cracking.

2. Acquisition of gluten network structure images by CLSM

CLSM measurements of the stained dough samples are performed by an eclipse Ti-U inverted microscope with an e-C1 plus confocal system using the most commonly Plan Apo20×/0.75 objectives. A laser with a wavelength of 543 nm is used for excitation, the emission is detected at 590 nm. 10 different images are taken of each dough sample with a resolution of 1024×1024 pixel and a size of 686×686 μm (for 20× objective), respectively.

3. Image processing and analysis by AngioTool64

3.1 Implementation details

CLSM images are converted to grayscale, blurred with a Gaussian filter whose protein thread diameter matches the apparent protein thread width, then thresholder to define the protein ROI using AngioTool64. The software skeletonizes the ROI to compute parameters of the gluten network, including protein area, protein percentage

area, total/average protein length, protein junctions, junctions density, and end-points. It also calculates lacunarity independently via box counting at multiple box sizes. From the direct outputs, derived metrics such as branching rate, end-point rate, and protein width quantify connectivity, breakage, and strand thickness of the gluten network.

3.2 Parameter setting

Gluten-thread diameter is set to 2 and 3 for images recorded with 20× objective, intensity low and high threshold to 15 and 255 for both magnifications, small particles were removed under 10 and the function “fill holes” is deactivated. Calibration is set to 1.49 pixel/μm for 20× objective.

Note: 1) each CLSM image is analyzed by AngioTool64 with the same settings and gluten-thread parameters to ensure a reproducible quantification of the protein network; 2) an overview of the CLSM workflow used to assess gluten network structures shown in Fig. 1.

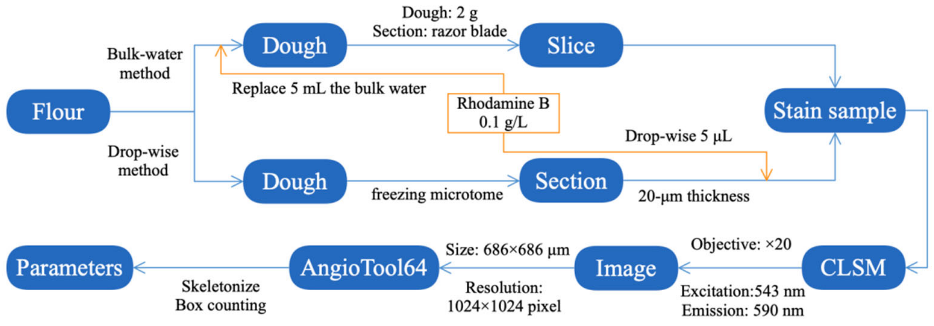

Fig. 1. Workflow of confocal laser scanning microscopy for assessing gluten network structure.

References

Bernklau, I., Lucas, L., Jekle, M., & Becker, T. (2016). Protein network analysis—A new approach for quantifying wheat dough microstructure. *Food Research International*, *89*, 812–819. https://doi.org/10.1016/j.foodres.2016.10.012

Lucas, I., Stauner, B., Jekle, M., & Becker, T. (2018). Staining methods for dough systems-Impact on microstructure and functionality. *LWT*, *88*, 139–145. https://doi.org/10.1016/j.lwt.2017.10.010

Zhang, M., Ma, M., Jia, R., Yang, T., Sun, Q., & Li, M. (2022). Delineating the dynamic transformation of gluten morphological distribution, structure, and aggregation behavior in noodle dough induced by mixing and resting. *Food Chemistry*, *386*, 132853. https://doi.org/10.1016/j.foodchem.2022.132853
