# Supplementary material for: Key Methodologies in Characterizing the Multi-Scale Structures of Gluten Proteins in Dough: A Comparative Review
Source: Biomolecules. 2026 Mar 3;16(3):382. doi: 10.3390/biom16030382 (PMC13023611; doi:10.3390/biom16030382)
Supplement: Supplementary file 1 [file biomolecules-16-00382-s001.zip › Supplementary File S16.pdf]

Supplementary material S16：

Analysis of the network structure of gluten proteins—scanning electron microscopy

Principle

The scanning electron microscope (SEM) examines the surface microscopic detail of solid specimens. A finely focused primary electron beam, emitted by the electron source, is raster scanned across the surface. Interactions between the beam and the specimen generate electron signals at each scan position, most commonly secondary electrons and backscattered electrons. These signals are collected, converted to electrical values, amplified, and synchronously mapped to image pixels. SEM presents intensity images where zero signal is displayed as black, intermediate signals as shades of grey and maximum signal as white. In conventional high vacuum operation, the specimen must be dry and electrically conductive, or it should be thinly coated to provide conductivity.

Apparatus

1. Glutomatic gluten washer: washing out starch and water-soluble fractions to isolate wet gluten.

2. Alpha 1-4 LD plus freeze dryer (Martin Christ, Osterode, Germany): freeze-drying dough/gluten samples for SEM observation.

3. Leica EM CPD300 critical point dryer: critical point drying of samples after chemical fixation and dehydration.

4. Phenom Pro10102 scanning electron microscope: SEM imaging of gluten network microstructure at accelerating voltage of 15 kV.

Reagents

1. Glutaraldehyde solution (5%, w/v): dissolved in 0.1 M phosphate buffer, pH 7.2. Primary fixation of dough/gluten samples before SEM observation.

2. Osmium tetroxide (OsO_4) solution (1%, w/v): dissolved in 0.1 M phosphate buffer, pH 7.2. Post-fixation to enhance contrast and stabilize network structures.

3. Phosphate buffer (0.1 M, pH 7.2); buffer solution for fixation and washing steps.

4. Ethanol series: concentration gradient: 30%, 50%, 70%, 80%, 95%, 100% (15 min each). Dehydration of samples before CPD drying.

5. NaCl solution (0.2 M): washing the dough to remove the starch particles.

6. α-amylase solution (Bacillus subtilis, 0.05%, w/v, in deionized water): starch is removed by treatment with it.

Procedure

1. Preparation of samples

1.1 Dough

The dough mixing was carried out on a Chopin MixoLab, using AOAC official methods of Analysis (17th edn), or the dough sample was prepared according to the experimental objectives.

1.2 Gluten

1.2.1 Washing method

Method 1: Gluten protein is obtained by washing out the starch granules from the dough using 0.2 M NaCl solution.

Method 2: The dough is then allowed to rest for 5 minutes to allow any residual stresses to relax, before being transferred to a glutomatic gluten washer. This washes out all the water-soluble and water-dispersible components, such as pentosans, starch and non-gluten proteins, by gently squeezing the dough under a stream of water, leaving behind the gluten as a water-insoluble, cohesive, viscoelastic mass.

1.2.1 Enzymatic method

Starch is removed by treatment with a solution of 0.05% α-amylase (Bacillus

subtilis) for 1h at room temperature, after holding the sample at 85℃ for a few seconds. Pieces of samples are stretched into membranes if needed and washed thoroughly in deionized water.

2. Drying process

For SEM, hydrated samples of dough are either freeze-dried, or critical-point-dried (CPD) after glutaraldehyde fixation.

2.1 Freeze-dried

The samples are cut into small cubes (approx. 2×12×12 mm) by razor blade and then freeze-dried for 48 h using an Alpha 1-4 LD plus freeze dryer.

2.2 Critical-point-dried

The cubic samples are initially fixed in 5% glutaraldehyde solution dissolved in phosphate buffer for 1 hour at room temperature. After fixation, the samples are washed three times with the same buffer to remove excess fixative. The cubic samples are then postfixed in 1% OsO_4 solution in phosphate buffer for 1 hour and washed with deionized water. Following fixation, the samples are dehydrated through a graded ethanol series (30%, 50%, 70%, 80%, 95%, 100%) for 15 minutes at each step. After ethanol dehydration, the samples are critical point dried in CO_2, using a Leica EM CPD300 critical point dryer.

3. Acquisition of gluten network structure images by SEM

The structure of the samples is characterized with the aid of a Phenom Pro10102 scanning electron microscope. After sputtering with gold-palladium alloy, the drying samples are subjected to blowing nitrogen to remove surface impurities and then mounted onto the specimen stage with conductive carbon tape. The exposed cross-sections of the samples were analyzed using the microscope operating at an accelerating voltage of 15 kV. The images were recorded at a magnification of 1000× (choose according to the experiment objective). Six different locations were selected for each sample to take SEM pictures.

4. Workflow diagram

An overview of the SEM workflow used to assess gluten network structures shown in Fig. 1.

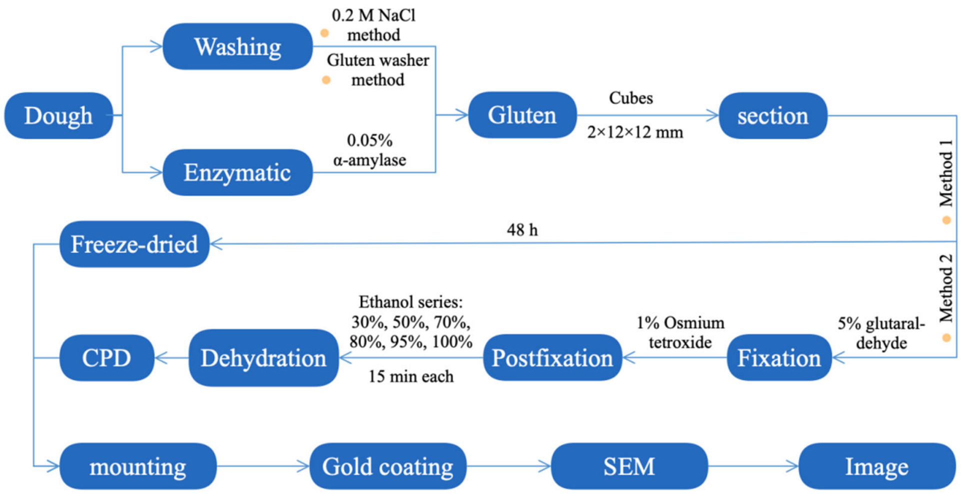

Fig. 1. Workflow of SEM for assessing gluten network structure.

References

Amend, T., & Belitz, H.-D. (1991). Microstructural studies of gluten and a hypothesis on dough formation. Food Structure, *10*(4), 277–288. https://digitalcommons.usu.edu/foodmicrostructure/vol10/iss4/1

Bache, I. C., & Donald, A. M. (1998). The Structure of the Gluten Network in Dough: A Study using Environmental Scanning Electron Microscopy. Journal of Cereal Science, 28(2), 127–133. https://doi.org/10.1006/jcrs.1997.0176

Yang, T., Wang, Y., Jiang, J., Wang, P., Zhong, Y., Zhou, Q., Wang, X., Cai, J., Huang, M., Jiang, D., Dai, T., & Cao, W. (2023). Influence of High-Molecular-Weight Glutenin Subunit on Components and Multiscale Structure of Gluten and Dough Quality in Soft Wheat. Journal of Agricultural and Food Chemistry, 71(12), 4943–4956. https://doi.org/10.1021/acs.jafc.2c07958

Zhang, M., Ma, M., Jia, R., Yang, T., Sun, Q., & Li, M. (2022). Delineating the dynamic transformation of gluten morphological distribution, structure, and aggregation behavior in noodle dough induced by mixing and resting. Food Chemistry, 386, 132853. https://doi.org/10.1016/j.foodchem.2022.132853
