# Supplementary material for: Key Methodologies in Characterizing the Multi-Scale Structures of Gluten Proteins in Dough: A Comparative Review
Source: Biomolecules. 2026 Mar 3;16(3):382. doi: 10.3390/biom16030382 (PMC13023611; doi:10.3390/biom16030382)
Supplement: Supplementary file 1 [file biomolecules-16-00382-s001.zip › Supplementary File S17.pdf]

Supplementary material S17:

Analysis of the network structure of gluten proteins—confocal Raman microscopy

Principle

Confocal Raman microscopy (CRM) combines the chemical sensitivity of Raman spectroscopy with the spatial resolution advantages of confocal microscopy. Thus, with a laser excitation, the spatial distribution of different components of a dough sample is recorded by CRM in terms of a chemical fingerprint image. From the resulting image, a precise localization of gluten network on a focal plane in the dough can be obtained as a monochrome image defined by the intensity of amide peak I (1600~1700 cm^{-1}).

Apparatus

1. WITec alpha300R confocal Raman microscope, equipped with an ultrasensitive EMCCD detector and 532 nm solid-state diode laser excitation source; used for Raman mapping acquisition to obtain Raman scattering signals of gluten, starch, and water in dough, and to realize preliminary imaging.

2. WITec Project software: used for data processing and imaging.

3. AngioTool64 software: quantitatively analyzes the characteristics of gluten network based on gluten skeleton images obtained by CRM.

Procedure

1. Preparation of the sample

Dough is prepared by mixing 500 g of wheat flour (Nisshin Seifun, crude protein 8.5%, ash 0.34%) with 160 g of Milli-Q water, followed by kneading using a mixer (Hobart, N50) for 20 min at 139 rpm to produce a wheat dough. The dough is cut into small cubes (approx. 2×5×5 mm) with a razor blade.

2. Instruments setup

Raman mapping of dough samples is carried out with a WITec alpha300R

microscope equipped with an ultrasensitive EMCCD detector and 532 nm solid-state diode laser excitation source.

The Raman scattered light from the sample is collected in the backscattering geometry onto a 600 groove/mm grating.

The 50× objective (0.55 numerical aperture) is used for confocal Raman imaging. A video stitching technique is first used to obtain a bigger field of view of the sample.

3. Raman mapping and spectrum acquisition

The sample is brought into focus under the microscope to select an appropriate area, and the selected area is then scanned to obtain a Raman scatter map. The image scan function is set to 100 scans per line and 100 lines per image, using 20 mW laser power and a 0.05 s integration time. For each dough component, the Raman spectrum is measured at 10 different points, with 20 accumulations of 1 s each per point, and the average spectrum is calculated for each component.

4. Data processing and imaging

The raw spectra are the average spectra of the scanned regions, and they are corrected using cosmic-ray removal (filter size 4, dynamic factor 8) and background subtraction (polynomial order 0).

The data from the area scans are processed in two different ways to produce images showing the spatial distribution of the single dough components (liquid water, starch, and gluten). These included the amide I band of proteins (1600-1700 cm^{-1}), the stretching vibrations of the starch carbon network (460-510 cm^{-1}), and the OH stretching band of water (3100-3680 cm^{-1}). By default, proteins are colored red and starch blue.

Method 1: By selecting the characteristic Raman band of gluten proteins, band integration is performed on the spectrum at each pixel to obtain monochrome images representing the intensity distribution of each component.

Method 2: The second method considered the full Raman spectra instead of single

bands. The Raman spectrum measured at each point of the sample is assumed to be a linear combination of the spectra of the single dough components. After performing a 3rd order polynomial background subtraction on all spectra, a multiple linear regression (MLR) is completed using the function Basis Analysis of the WITec Project software (release 2.10, WITec GmbH, Ulm, Germany). The assessed regression coefficients were used as indicators of the concentration of the individual dough components, and corresponding monochrome images were generated.

Finally, the monochrome images are combined into a color image using the True Component Analysis and Filter Manager functions in WITec software, with each color representing a component, thereby visually displaying the distribution of gluten protein.

5. Workflow diagram

An overview of the CRM workflow used to assess gluten network structures is shown in Fig. 1.

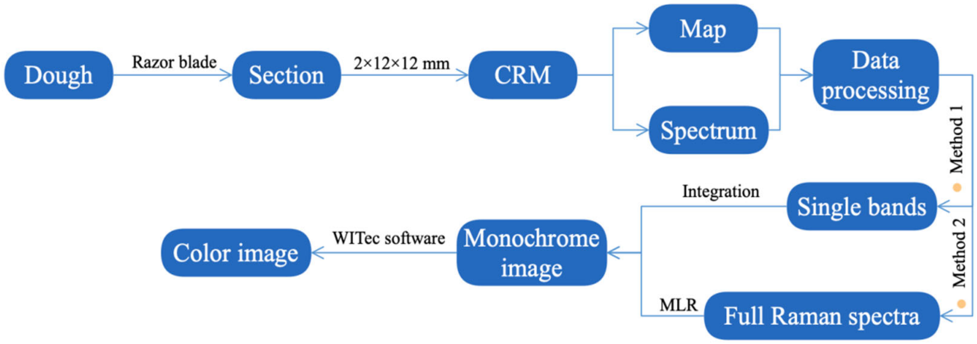

Fig. 1. Workflow of CRM for assessing gluten network structure.

References

Alinovi, M., Mucchetti, G., Andersen, U., Rovers, T. A. M., Mikkelsen, B., Wiking, L., & Corredig, M. (2020). Applicability of Confocal Raman Microscopy to Observe Microstructural Modifications of Cream Cheeses as Influenced by Freezing. Foods, 9(5), 679. https://doi.org/10.3390/foods9050679

He, W., Wang, M., Li, M., Zhong, Z., Chen, H., Xi, S., Luan, Z., Li, C., & Zhang, X. (2022). Confocal Raman microscopy for assessing effects of preservation methods on symbiotic deep-sea mussel gills. Frontiers in Marine

Science, 9, 1077595. https://doi.org/10.3389/fmars.2022.1077595

Huen, J., Weikusat, C., Bayer-Giraldi, M., Weikusat, I., Ringer, L., & Lösche, K. (2014). Confocal Raman microscopy of frozen bread dough. Journal of Cereal Science, 60(3), 555–560. https://doi.org/10.1016/j.jcs.2014.07.012
