# Supplementary material for: Key Methodologies in Characterizing the Multi-Scale Structures of Gluten Proteins in Dough: A Comparative Review
Source: Biomolecules. 2026 Mar 3;16(3):382. doi: 10.3390/biom16030382 (PMC13023611; doi:10.3390/biom16030382)
Supplement: Supplementary file 1 [file biomolecules-16-00382-s001.zip › Supplementary File S18.pdf]

Supplementary material S18：

Analysis of the network structure of gluten proteins—two-photon excitation microscopy

Principle

Conventional microscopy is limited to providing two-dimensional section images, whereas optical clearing reduces light scattering by matching the refractive indices within the sample, thereby rendering it transparent and enabling deep tissue imaging. Using a sodium salicylate-based clearing reagent (SoROCS), dough can be rendered transparent within a few days without compromising the integrity of the gluten structure. Following fluorescent labeling, two-photon excitation microscopy (2PEM) enables millimeter-scale three-dimensional (3D) visualization with submicron resolution, while confocal laser scanning microscopy (CLSM) provides high-resolution 3D imaging at relatively shallow depths (~600 μm). Furthermore, quantitative analysis can be performed by importing optical section images and reconstructed 3D datasets into AngioTool software, which allows quantitative analysis by the extraction of key structural parameters.

Apparatus

1. Two-photon excitation microscopy: used for 3D imaging of gluten networks in SoROCS cleared dough samples.

2. ×25 immersion objective lens for SoROCS: numerical aperture (NA)=1.00, working distance (WD)=8 mm, refractive index (RI) adaptation range=1.41-1.52; with a correction collar); used with 2PEM to focus the laser on SoROCS-treated samples and collect fluorescence signals of labelled gluten.

3. Inverted confocal laser scanning microscopy (CLSM) system: used for imaging gluten structures in SoROCS-cleared samples, providing an alternative imaging method to 2PEM for researchers with limited access.

4. Refractometer: used to determine the refractive index (RI) of SoROCS at 30 °C,

ensuring SoROCS meets the required RI (1.455 at room temperature) for optical clearing.

5. Protein network analysis software (AngioTool64 Ver. 0.6a, National Cancer Institute, National Institute of Health, USA); used to analyze the characteristics of gluten skeletal structure images obtained from CLSM and 2PEM, quantifying gluten network parameters.

6. Imaging control software; used to control parameters of 2PEM or CLSM (such as laser power, detector sensitivity), collect fluorescence image data, and generate 2D slices and 3D stacked images.

7. Laser source: used to provide specific wavelengths of laser for 2PEM, with 940 nm for exciting Thiolite™ Green (TG) and 840 nm for exciting Alexa Fluor™ 633 C_5 Maleimide (AF 633).

8. Diode lasers: used to provide specific wavelengths of laser for CLSM, with 473 nm for exciting TG and 559 nm for exciting AF 633.

Reagents

1. Sodium salicylate: dissolved completely in Milli-Q water under stirring and heating at 70 °C to form a 4 M sodium salicylate solution; core component of SoROCS, promoting starch gelatinization to reduce light scattering in wheat-based samples, contributing to optical clearing.

2. Triton X-100: added to 4 M sodium salicylate solution to a concentration of 0.1% (w/w); detergent for lipids in wheat-based samples, removing lipids to further reduce light scattering, assisting SoROCS in improving sample transparency.

3. SoROCS (optical clearing reagent): specification: composed of 4 M sodium salicylate and 0.1% (w/w) Triton X-100, pH=7.5, RI=1.455 at room temperature, almost colorless; preparation: refer to the preparation methods of sodium salicylate and Triton X-100 above; function: making wheat-based products (such as dough) transparent within a few days, reducing light scattering to enable deep 3D imaging of

gluten.

4. Thiolite™ Green: specification: preparation for fluorescent labelling: 10 mM TG stock solution dissolved in DMSO was added to SoROCS to yield a final concentration of 0.0005% (w/w); specifically reacting with thiol groups (cysteine residues) on gluten proteins to label gluten, enabling fluorescence imaging of gluten networks.

5. Alexa Fluor™ 633 C_5 Maleimide: 10 mM AF 633 stock solution dissolved in DMSO was added to SoROCS to yield a final concentration of 0.0002% (w/w); specifically labeling gluten proteins, cooperating with 2PEM or CLSM to realize fluorescence imaging of gluten networks.

6. Dimethyl sulfoxide: solvent for dissolving TG and AF 633, preparing their stock solutions.

7. Paraformaldehyde solution (PFA, 4%, w/w): dissolving PFA in PBS to reach 4% (w/w) concentration; fixing dough samples at 30 °C for 1.5 h, maintaining the original structure of gluten networks and preventing sample degradation.

8. Dulbecco's phosphate-buffered saline (D-PBS): washing fixed dough samples twice to remove residual PFA, avoiding interference with subsequent optical clearing.

Procedure

1. Preparation of SoROCS

Take an appropriate amount of sodium salicylate and add it to ultrapure water. Under stirring and heating at 70 °C, dissolve the sodium salicylate completely to prepare a 4 M sodium salicylate solution.

Add Triton X-100 to the 4 M sodium salicylate solution, and stir uniformly to make the final concentration of Triton X-100 reach 0.1% (w/w).

Use a refractometer to determine the refractive index (RI) of the prepared solution at 30 °C, ensuring the RI is 1.455 (room temperature RI standard) and the pH is 7.5; the

resulting solution is SoROCS.

For fluorescent labelling of gluten: Dissolve Thiolite^{TM} Green (TG, AAT Bioquest, Cat. No. 21508) in DMSO (Wako Chemicals, Cat. No. 043-07216) to prepare a 10 mM stock solution, then add it to SoROCS to a final concentration of 0.0005% (w/w); dissolve Alexa Fluo^{TM} 633 C_5 Maleimide (AF 633, Invitrogen, Cat. No. A20342) in DMSO to prepare a 10 mM stock solution, then add it to SoROCS to a final concentration of 0.0002% (w/w).

2. Preparation of dough

Weigh 500 g of wheat flour (Nisshin Seifun, crude protein 8.5%, ash 0.34%) and 160 g of Milli-Q water.

Add the wheat flour and ultrapure water to a mixer, and knead at 139 rpm for 20 minutes to form a uniform wheat dough.

Use a razor blade to cut the kneaded wheat dough into small cubes with an approximate size of 0.7×1.8×1.8 mm, which are the dough samples to be treated.

3. Fixation and optical clearing

Place the dough cubes into 4% (w/w) paraformaldehyde (PFA) solution (dissolved in PBS) and fix at 30 °C for 1.5 h to maintain the structure of gluten networks.

After fixation, take out the dough samples and wash them twice with Dulbecco's phosphate-buffered saline (D-PBS) to remove residual PFA.

Transfer the washed dough samples into the prepared SoROCS, place the system at 30 °C, and shake for 3 days to realize optical clearing of the samples.

4. Acquisition of gluten network structure images by 2PEM or CLSM

4.1 Two-photon excitation microscopy imaging

Select an upright or inverted 2PEM system, and install a ×25 immersion objective lens for SoROCS (Olympus, XLSLPLN25XGMP; NA=1.00, WD=8 mm, RI=1.41–1.52).

Place the SoROCS-cleared dough sample under the objective lens, and enable the brightness compensation function in the z direction to adjust detector sensitivity and laser power according to imaging depth.

Turn on the laser source: use a 940 nm laser to excite TG-labelled gluten, and an 840 nm laser to excite AF 633-labelled gluten.

Use a normal photomultiplier tube (PMT) to quantify the fluorescence signal of AF-labelled gluten, and a gallium arsenide phosphide (GaAsP) PMT to quantify the fluorescence signal of TG-labelled gluten.

Operate the imaging control software to collect fluorescence images and generate 2D optical sections or 3D stacked images of gluten networks.

4.2 Confocal laser scanning microscopy imaging

Use an inverted CLSM system and install a ×20 dry objective lens.

Put the SoROCS cleared dough sample on the sample stage of the CLSM and use the brightness compensation function in the z direction to adjust detector sensitivity and laser power to adapt to different imaging depths.

Turn on the diode lasers: use the 473 nm laser to excite TG-labelled gluten, and the 559 nm laser to excite AF-labelled gluten.

Run the imaging control software to perform imaging operations, collect fluorescence images of gluten at different depths (up to 600 μm, limited by the objective lens WD), and record imaging data.

5. Protein Network Analysis

Import the gluten structure images obtained from CLSM and 2PEM into the protein network analysis software.

Preprocess the images: convert the color images to grayscale images first, then apply a Gaussian filter (with filter diameter matching the apparent width of gluten threads) to blur the images and perform thresholding to define the region of interest

(ROI) of the image.

Set software parameters to ensure reproducible quantification: set vessel thickness to 8, intensity low threshold to 0, and high threshold to 255, remove small particles with a size less than 30 pixels, and deactivate the "fill holes" function.

Use the software to skeletonize the gluten ROI, and compute gluten network parameters, including gluten area, gluten percentage area, total gluten length, average gluten length, gluten junctions, junction density, end points, end points rate, and mean E lacunarity.

Derive additional metrics (such as branching rate, gluten width) from the direct output parameters to quantify the connectivity, breakage degree, and strand thickness of the gluten network, and record and analyze the data.

5. Workflow diagram

An overview of the 2PEM or CLSM workflow used to assess 3D gluten network structures is shown in Fig. 1.

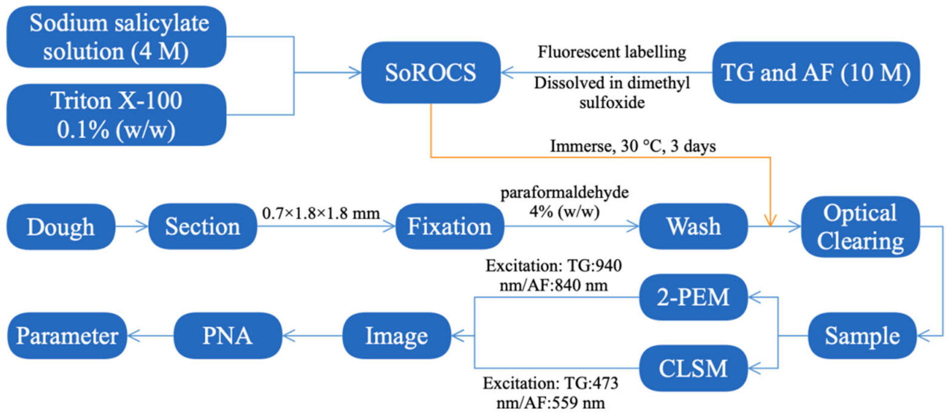

Fig. 1. Workflow of 2PEM or CLSM for assessing 3D gluten network structure.

References

Bernklau, I., Lucas, L., Jekle, M., & Becker, T. (2016). Protein network analysis—A new approach for quantifying wheat dough microstructure. *Food Research International*, *89*, 812–819. https://doi.org/10.1016/j.foodres.2016.10.012

Ogawa, T., & Matsumura, Y. (2021). Revealing the 3D structure of gluten in wheat dough by optical clearing imaging. Nature Communications, 12(1), 1708. https://doi.org/10.1038/s41467-021-22019-0
