# Supplementary material for: Key Methodologies in Characterizing the Multi-Scale Structures of Gluten Proteins in Dough: A Comparative Review
Source: Biomolecules. 2026 Mar 3;16(3):382. doi: 10.3390/biom16030382 (PMC13023611; doi:10.3390/biom16030382)
Supplement: Supplementary file 1 [file biomolecules-16-00382-s001.zip › Supplementary File S1.pdf]

Supplementary material S1:

Fractionation of gluten fractions—Osborne/Sequential extraction method

Principle

The Osborne or sequential extraction method relies on the differential solubility of proteins in various solvents. Proteins are fractionated by progressively altering solvent conditions.

Apparatus

1. UltraTurrax homogenizer: used to process mixtures into homogeneous, stable, and well-dispersed solutions.

2. Eppendorf 5810R centrifuge: used to separate supernatant and residue after extraction to obtain target protein fractions.

3. Freeze dryer: used for freeze-drying extractives.

4. Mixer: used to knead wheat dough at 139 rpm for 20 min.

Reagents

1. Sodium phosphate buffer (Na_2HPO_4/KH_2PO_4): prepared as a 0.067 mol/L solution, adjusted to pH 7.6; used for Osborne extraction buffer.

2. Sodium chloride: prepared as a 0.5 mol/L solution; used in phosphate buffer for albumin/globulin extraction.

3. Ethanol: prepared as a 60% aqueous solution; used for gliadin extraction.

4. 1-Propanol: prepared as a 50% solution; used in glutenin extraction buffer.

5. Dithiothreitol (DTT): prepared as 1% (w/v) in extraction buffer; used as a reducing agent for glutenin extraction.

6. Sodium iodide (NaI): prepared as a 0.3 mol/L solution with 7.5% 1-propanol; used for sequential extraction of gliadin.

7. Ammonium Acetate-Methanol (NH4Ac-MeOH) buffer solution: adding four volumes of cold (4 °C) 0.1 M ammonium acetate in 100% methanol; used for the precipitation of proteins in the supernatant.

8. SDS-DTT-TRIS buffer solution: 0.4 mL 2% SDS and 25 mM DTT in 25 mM TRIS, pH 8.0; used for extracting glutenin from freeze-dried pellet 1 in the Sequential Extraction method.

Procedure

1. Preparation of samples

Dough is prepared by mixing 500 g of wheat flour (Nisshin Seifun, crude protein 8.5%, ash 0.34%) with 160 g of deionized water, followed by kneading using a mixer for 20 min at 139 rpm to produce a wheat dough. The dough is freeze-dried and then ground through a 100-mesh sieve.

2. Osborne method

Add 60 mL of 0.067 mol/L Na_2HPO_4/KH_2PO_4 buffer (pH 7.5) and 0.5 mol/L NaCl to 15g of dough powder. Use an UltraTurrax homogenizer to mix and extract at 22°C and 12,400 r/min for 5 min. Centrifuge the suspension (25 min, 3750×g, 22°C) to obtain the supernatant. Repeat the process three times and combine the supernatants to obtain albumin/globulin.

Extract gliadin from the residue three times with 60 mL of 60% ethanol using the same process as above.

Next, extract glutenin from the residue twice with 60 mL of 50% 1-propanol, 0.05 mol/L Na_2HPO_4/KH_2PO_4 (pH 7.5), and 1% DTT under a nitrogen atmosphere using the same process as above.

3. Sequential Extraction method

Take 100 mg of the dough powder sample and extract it with 1 ml of 0.3 M NaI, 7.5% 1-propanol (NaI-propanol) solution. Centrifuge at 4500×*g* for 10 min in an

Eppendorf 5810R centrifuge. After two extractions, the supernatant fractions are pooled, precipitated with four volumes of cold (-20 °C) NH_4Ac-MeOH, and stored at -20 °C for at least 48 hours. Then centrifuge under the above conditions to obtain the gliadin fraction. The residue (pellet 1) is saved.

After precipitation of the gliadin with NH_4Ac-MeOH, the remaining proteins are recovered from the NaI supernatant fluid by precipitating the proteins with four volumes of acetone, storing at -20 °C overnight, and centrifuging as above to produce the albumin/globulin fraction.

To prepare the glutenin fraction, pellet 1 is freeze-dried, powdered, and then extracted with 0.4 mL of SDS-DTT-TRIS buffer per 100 mg of sample and centrifuged as above. After two extractions, combine the dissolved proteins and precipitate with NH_4Ac-MeOH to obtain the glutenin fraction.

The extracted protein fractions are immediately freeze-dried and stored at -80 °C.

4. Workflow diagram

An overview of the Osborne or sequential extraction method workflow is shown in Fig. 1.

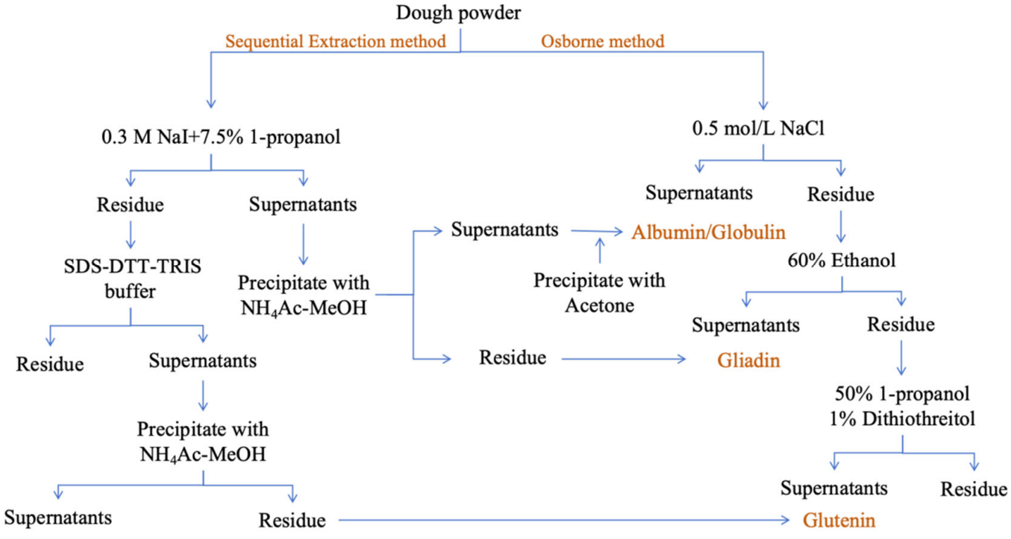

Fig. 1. Workflow of the Osborne or sequential extraction method for fractionation of gluten fractions.

References

Dupont, F. M., Chan, R., Lopez, R., & Vensel, W. H. (2005). Sequential extraction and quantitative recovery of gliadins, glutenins, and other proteins from small samples of wheat flour. *Journal of Agricultural and Food Chemistry*, *53*(5), 1575–1584. https://doi.org/10.1021/jf048697l

Osborne T. B. (1907). The Proteins of the Wheat Kernel. *Science*, *26,* 865–865. https://doi.org/10.1126/science.26.677.865

Schalk, K., Lexhaller, B., Koehler, P., & Scherf, K. A. (2017). Isolation and characterization of gluten protein types from wheat, rye, barley and oats for use as reference materials. *PLOS ONE*, *12*(2), e0172819. https://doi.org/10.1371/journal.pone.0172819
