# Supplementary material for: Key Methodologies in Characterizing the Multi-Scale Structures of Gluten Proteins in Dough: A Comparative Review
Source: Biomolecules. 2026 Mar 3;16(3):382. doi: 10.3390/biom16030382 (PMC13023611; doi:10.3390/biom16030382)
Supplement: Supplementary file 1 [file biomolecules-16-00382-s001.zip › Supplementary File S2.pdf]

Supplementary material S2:

Analysis of monomeric/subunit composition of gluten fractions—sodium dodecyl sulfate polyacrylamide gel electrophoresis

Principle

Sodium dodecyl sulfate (SDS) molecules bind to denatured proteins at a consistent ratio, which effectively masks the inherent charge heterogeneity of protein molecules and results in a molecular size-directed separation for them. After the operation procedures such as electrophoretic migration and staining, the monomers or subunits of proteins with significantly deferred molecular weight stand as independent bands in the running gel, which allow their identity authentication, molecular weight measurement, and abundance determination via the locations and intensities (grayscales) of these bands in reference to standard markers.

Apparatus

1. Vertical electrophoresis apparatus: used for SDS-PAGE analysis with 12% separating gel and 5% stacking gel.

2. Gel scanner: used for scanning protein gels to obtain clear gel images for subsequent analysis.

3. ImageJ software: used for data analysis of SDS-PAGE gels, including quantification of protein bands and calculation of molecular weights.

Reagents

1. Reduced loading buffer: containing Tris-HCl (0.1 M, pH 6.8), glycerol (10%, v/v), bromophenol blue (0.1%, w/v), SDS (2%, w/v), and β-mercaptoethanol (5%, w/v).

2. Unreduced loading buffer: containing Tris-HCl (0.1 M, pH 6.8), glycerol (10%, v/v), bromophenol blue (0.1%, w/v), SDS (2%, w/v). It does not contain reducing agents, thus preserving disulfide bonds in proteins.

3. Isopropanol aqueous solution (50%, v/v); used for extracting gliadins from dough powder, promoting the dissolution of gliadins.

4. Glutenins extraction solution A: isopropanol（50%, v/v), Tris-HCl (0.08 M, pH 8.0), dithiothreitol (DTT, 1%, w/v).

5. Glutenins extraction solution B: isopropanol（50%, v/v), Tris-HCl (0.08 M, pH 8.0), 1.4% 4-Vinylpyridine.

6. 12% separating gel formula: 4.0 mL of 30% Acrylamide/Bisacrylamide (29:1); 2.5 mL of 1.5 M Tris-HCl (pH 8.8); 3.4 mL of Deionized water; 0.1 mL of 10% (w/v) SDS; 0.1 mL of Freshly prepared 10% (w/v) ammonium persulfate (APS), 4.0 μL of Tetramethylethylenediamine (TEMED). It forms a gel with small pores for separating proteins based on molecular weight.

7. 5% concentration gel formula (5 mL/gel): 0.83 mL of 30% Acrylamide/Bis (29:1); 0.63 mL of 0.5 M Tris-HCl (pH 8.8); 3.40 mL of Deionized water; 0.05 mL of 10% (w/v) SDS; 0.05 mL of Freshly prepared 10% (w/v) APS, 4.00 μL of TEMED. It forms a gel with large pores to concentrate proteins into narrow bands before they enter the separating gel.

8. 10× Tris-Glycine SDS electrophoresis buffer: Tris-base (0.25 M), glycine (1.92 M, w/v), and SDS (1%, w/v). When diluted to 1×, it serves as the running buffer for SDS-PAGE, conducting current and maintaining the pH stability (around 8.3-8.5) of the electrophoresis system.

9. Protein marker: serves as a molecular weight reference standard to estimate the molecular weight of proteins in samples.

10. Coomassie Brilliant Blue R-250: staining protein bands blue for visualization.

11. Destained (acetic acid: methanol: distilled water=10:45:45, v/v/v); Removes unbound Coomassie Brilliant Blue R-250 dye from the gel, reducing background staining and making protein bands clearer.

Procedure

1. Sample preparation

1.1 Dough

Dough is prepared by mixing 500 g of wheat flour (Nisshin Seifun, crude protein 8.5%, ash 0.34%) with 160 g of deionized water, followed by kneading using a mixer for 20 min at 139 rpm to produce a wheat dough. The dough is freeze-dried, then ground into powder and passed through a 100-mesh sieve.

1.2 Extraction of reduced gluten proteins

Weigh 30 mg of dough powder into a 5 mL centrifuge tube, and add 2 mL of reduced loading buffer. After thorough mixing, the mixture is continuously shaken for 1 hour, followed by centrifugation (12,000×*g,* 10 min). The supernatant is collected, heated in boiling water at 100°C for 5 min, allowed it to room temperature, and loaded onto the SDS-PAGE gel for electrophoresis.

1.3 Extraction of unreduced gluten proteins

Weigh 30 mg of dough powder into a 5 mL centrifuge tube, and add 2 mL of unreduced loading buffer. After thorough mixing, the mixture is continuously shaken for 1 hour, followed by centrifugation (12,000×*g,* 10 min). The supernatant is collected, heated in boiling water at 100 °C for 5 min, allowed it to room temperature, and loaded onto the SDS-PAGE gel for electrophoresis.

1.4 Extraction of gliadins and glutenins

Weigh 100 mg of dough powder, add 1 mL of 50% isopropanol aqueous solution, mix well with a vortex shaker, and place in a shaker. Shake at 65 °C for 30 min at 200 rpm/min. After cooling at room temperature, centrifuge (10,000×*g*, 20 °C, 10 min) and collect the supernatant. Repeat the above steps again for the precipitation section, and combine the two supernatants to obtain the gliadins.

Add 1 mL of glutenins extraction solution A to the precipitate. Place in a shaker and shake at 65 °C at 200 r/min for 30 min. Add 1 mL of glutenins extraction solution B and continue shaking under the above conditions for 15 min. Centrifuge (10,000×*g*,

20 ℃, 10 min) and collect the supernatant, which is the glutenins.

Mix the gliadins and glutenins extract with the reduced loading buffer in a 1:1 ratio, heat in a boiling water bath for 5 min, and allow it to room temperature for sample loading

2. SDS-PAGE

2.1 Gel preparation

Prepare polyacrylamide gel according to the formula of 12% separating gel and 5% concentrated gel.

2.2 Gel casting

Install the vertical electrophoresis plate of the electrophoresis instrument according to the experimental instructions, ensuring good sealing at the bottom of the electrophoresis plate to prevent liquid leakage.

Quickly pour the 12% separation gel prepared in advance into the gel device, reserve the space required for pouring the concentrated gel to sample (~2 cm), evenly cover a layer of ethanol (20%, v/v) on the separation gel layer with a liquid transfer gun, ensure that the separation gel surface is flat, and place it for 30 min at room temperature to make the separation gel fully polymerize.

Pour out the water layer on the separation gel and use filter paper to absorb the remaining moisture. Add the prepared 5% concentrated gel onto the top of the separation gel, gently insert the electrophoresis comb, being careful not to introduce bubbles, and polymerize at room temperature for 1 hour to fully polymerize the concentrated gel.

Fix the gel device into the electrophoresis tank, add appropriate Tris-Glycine SDS electrophoresis buffer diluted ten times into the upper tank and the lower tank, and the buffer solution should cover the gel, and carefully pull out the electrophoresis comb vertically upwards.

2.3 Sample loading

A mixture of seven proteins (M_r 6,500-200,000 Da) is used as a marker. According to the predetermined sequence, the sample size for each well is 6 μL (15 lanes, adjust according to the actual protein concentration). Under room temperature conditions, 120V constant pressure electrophoresis takes about 1.5 hours. The bromophenol blue indicator has reached the bottom of the separation gel, indicating the end of separation. (Note: the optimal electrophoresis time should be selected based on the molecular weight of the sample protein.)

2.4 Staining and destaining

After electrophoresis, take out the rubber plate, remove the concentrated gel, carefully peel off the separation gel, wash the electrophoresis buffer from the gel with distilled water, and cut a small notch on the gel to mark the sampling sequence.

Put the gel into the culture dish, add an appropriate amount of Coomassie brilliant blue (CBB) R-250 dye solution, put it on the shaking table for dyeing for 1 h, then wash it with destainer for 2-3 times, and shake it slowly in distilled water for overnight decolorization.

2.5 Image acquisition and processing

The gels are scanned, the images converted to grayscale, the lanes of interest plotted as x/y-diagrams and the peaks integrated using ImageJ open source software.

3. Workflow diagram

An overview of the SDS-PAGE workflow used for analyzing the monomeric/subunit composition of gluten fractions is shown in Fig. 1.

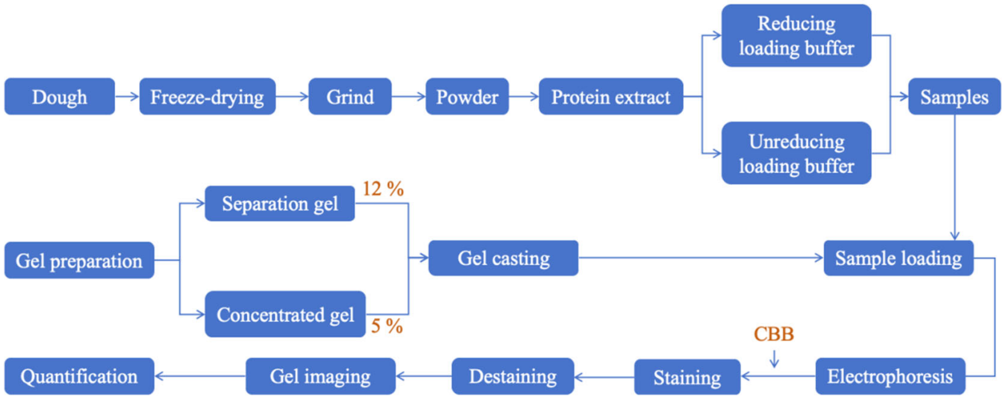

Fig. 1. Workflow of SDS-PAGE for the analysis of monomeric/subunit composition of gluten fractions.

Result presentation

1. Relative molecular weight (M_r) determination

Measure the electroretic mobility distances of standard proteins, the target protein, and the dye. Calculate the relative mobility rate using Formula (1). Determine the relative molecular mass of the protein based on the standard curve.

2. Intensity of quantitative electrophoresis band: the intensity of the gel electrophoresis band can be quantified by using ImageJ for grayscale analysis.

$$\text{Relative mobility rate} = \frac{\text{Sample mobility distance (cm)}}{\text{Dye mobility distance (cm)}} \quad (1)$$

References

Candiano, G., Bruschi, M., Musante, L., Santucci, L., Ghiggeri, G. M., Carnemolla, B., Orecchia, P., Zardi, L., & Righetti, P. G. (2004). Blue silver: A very sensitive colloidal Coomassie G-250 staining for proteome analysis. *Electrophoresis*, *25*(8), 1327-1333. https://doi.org/10.1002/elps.200305844

Rahaman, T., Vasiljevic, T., & Ramchandran, L. (2016). Shear, heat and pH induced conformational changes of wheat gluten-Impact on antigenicity. *Food Chemistry*, 196, 180-188. https://doi.org/10.1016/j.foodchem.2015.09.041

Schalk, K., Lexhaller, B., Koehler, P., & Scherf, K. A. (2017). Isolation and characterization of gluten protein types from wheat, rye, barley and oats for use as reference materials. *PLOS ONE*, *12*(2), e0172819. https://doi.org/10.1371/journal.pone.0172819

Schirmer, T. M., Ludwig, C., & Scherf, K. A. (2023). Proteomic characterization of wheat protein fractions taken at different baking conditions. *Journal of Agricultural and Food Chemistry*, *71*(32), 12899 – 12909. https://doi.org/10.1021/acs.jafc.3c02100
