# Supplementary material for: Key Methodologies in Characterizing the Multi-Scale Structures of Gluten Proteins in Dough: A Comparative Review
Source: Biomolecules. 2026 Mar 3;16(3):382. doi: 10.3390/biom16030382 (PMC13023611; doi:10.3390/biom16030382)
Supplement: Supplementary file 1 [file biomolecules-16-00382-s001.zip › Supplementary File S3.pdf]

Supplementary material S3：

Analysis of monomeric/subunit composition of gluten fractions—2-dimensional gel electrophoresis

Principle

2-dimensional gel electrophoresis (2-DE) includes two sequentially-arranged separation processes in different dimensions: the first dimension one realizes by isoelectric focusing (IEF), which allow the satisfied separation of protein molecules according to their isoelectric points, and in the second dimension, sodium dodecyl sulfate polyacrylamide gel electrophoresis (SDS-PAGE) is performed, which fulfills perpendicularly to the first dimension and further separates protein molecules in the light of their molecular weight. Under these circumstances, a high-resolution 2D protein map is concluded from 2-DE.

Apparatus

1. Mini Protean II Tube Cell. The mini two-dimensional electrophoresis cell is used for high-resolution separation of proteins.

2. Gel scanner: used for scanning protein gels to obtain clear gel images for subsequent analysis.

3. ImageJ software: used for basic gel image analysis, such as the observation and simple quantification of gel spots.

4. Progenesis SameSpots Ver. 4.5. Software: used for proteomic research in 2-DE, such as inter-gel protein spot matching, differential expression analysis, and database construction to compare protein profiles and analyze differential protein spots.

Reagents

1. NaCl solution (2%, w/v): used to wash out the starch from the dough to obtain the wet gluten.

2. SD buffer (0.3% SDS, 15 mM dithiothreitol): used for extracting gluten protein.

3. UCD buffer (8 M Urea, 4% CHAPS, 60 mM DTT): used for extracting gluten protein.

4. Trichloroacetic Acid (6.1 N, w/v): used for protein purification.

5. Acetone (-20 °C): used for protein washing.

6. Urea buffer (9 M urea, 4% Nonidet P-40, 1% DTT, and 2% 3-10 Iso-Dalt Grade Servalyts): used for protein lysis and rehydration buffers.

7. IEF gels: contained 9.2 M urea, 4% acrylamide/Bisacrylamide (Bis), 2% Nonidet P-40, 2% 3-10 Iso-Dalt Grade Servalyts, 0.015% ammonium persulfate, and 0.125% TEMED.

8. Sulfuric acid (0.2%, v/v): used for the upper electrode (anode) buffer.

9. Ethanolamine (0.5%, v/v): used for the lower electrode (cathode) buffer.

10. Urea (5 M, w/v): used to overlie IEF gels.

11. Equilibration buffer: 2.3% SDS, 10% glycerol, 0.05% dithiothreitol, 62.5 mM Tris-HCl, pH 6.8.

12. Protein marker: serves as a molecular weight reference standard to estimate the molecular weight of proteins in samples.

13. 10× Tris-Glycine SDS running buffer: Tris-base (0.25 M), glycine (1.92 M, w/v), and SDS (1%, w/v). When diluted to 1×, it serves as the running buffer for SDS-PAGE, conducting current and maintaining the pH stability (around 8.3-8.5) of the electrophoresis system.

14. SDS-PAGE precast gels: 12% separating gel formula and 5% concentration gel formula. Separating gel forms a gel with small pores for separating proteins based on molecular weight. A concentration gel forms a gel with large pores to concentrate proteins into narrow bands before they enter the separating gel.

15. Ammonium sulfate (20%, w/v): used for storing the destaining gel.

Procedure

1. Protein preparation

Dough is prepared by mixing 500 g of wheat flour (Nisshin Seifun, crude protein 8.5%, ash 0.34%) with 160 g of deionized water, followed by kneading using a mixer for 20 min at 139 rpm to produce a wheat dough. Fresh dough is ished with a 2% NaCl solution until the ishings became clear. This process is continued until the water used to rinse the dough no longer turns blue when tested with iodine solution, yielding wet gluten. The isolated wet gluten is freeze-dried, then ground and passed through an 80-mesh sieve to obtain gluten protein powder.

Gluten protein powder (10 mg) is dissolved in 1.5 mL of SD buffer. After centrifugation (11,600×*g*, 4 °C, 30 min), the precipitate is placed in a 50 mL centrifuge tube, and 10 mL of UCD buffer with strong dissociation is selected for the separation of all the gluten proteins. After full shaking, the samples are kept on ice and shaken every 5 min for a total of 6 times. After centrifugation (11,600×*g*, 4 °C, 30 min), the supernatant is aspirated and transferred to a 15 mL centrifuge tube.

2. Protein purification

The total protein fraction is precipitated with TCA to remove SDS and salts that interfere with isoelectric focusing. One volume of 6.1 N TCA is added to 4 volumes of the sample, and incubated on ice for 10 min. Samples are centrifuged (11,600×*g*, 4 °C, 15 min) and the supernate discarded. The pellets are rinsed 3 times with 200 μL acetone. The pellet suspensions are centrifuged (11,600×*g*, 4 °C, 15 min) after each rinse, and the final pellet is air dried at room temperature.

3. Protein quantification

The protein amount in the total protein fraction is determined by the method of Lowry. Urea buffer is added to the dried pellets of protein fraction. Samples are incubated at room temperature for 1 h in a microtube shaker and are then centrifuged (11,600×g, 25 °C, 10 min), and the supernatants are retained.

4. Isoelectric focusing (IEF)

IEF is performed using a Mini Protean II Tube Cell. The upper electrode (anode) buffer is sulfuric acid, and the lower electrode buffer (cathode) is ethanolamine. Because the anode buffer is acidic, the wires from the electrophoresis cell are reversed at the power supply. The gels are pre-focused at 200 V for 10 min, 300 V for 15 min, and 400 V for 15 min. Protein (15 μg) is loaded onto the IEF gels and overlain with 5 M urea. IEF gels are run at 500 V for 10 min and then increased to 750 V for 1 h.

5. Gel strip equilibration

Gels are extruded into tubes containing equilibration buffer. Gels are frozen immediately on dry ice and stored at -80 °C.

6. Sodium dodecyl sulfate polyacrylamide gel electrophoresis (SDS-PAGE)

Proteins are separated in the second dimension by SDS gel electrophoresis using an XCell SureLock Mini-Cell electrophoresis system. IEF gels are thawed and immediately placed on top of SDS-PAGE precast gels, 1 mm thick with a 2-D well. The IEF gel is overlain with 45 μL of equilibration buffer. Protein Standard (4 μL) is loaded into the standard well. The SDS gels are run with SDS running buffer for 50 min at 200 V.

7. Staining, and destaining

Gels are stained overnight with Coomassie brilliant blue (CBB) R-250, destained in water for 2 h at room temperature, and stored at 4 °C in 20% ammonium sulfate.

8. Gel image acquisition and analysis

All 2-DE gels are digitized with a calibrated scanner at 310 dpi. The protein spot pattern of two replicate 2-DE gels of each fraction is aligned and matched using Progenesis software. Spot volumes of proteins are determined by the Progenesis software for each gel.

In addition, a simpler approach can also be chosen. The gels are scanned, the

images converted to grayscale, the lanes of interest plotted as x/y-diagrams, and the peaks integrated using ImageJ open-source software.

9. Workflow diagram

An overview of the 2-DE workflow used for analyzing the monomeric/subunit composition of gluten fractions is shown in Fig. 1.

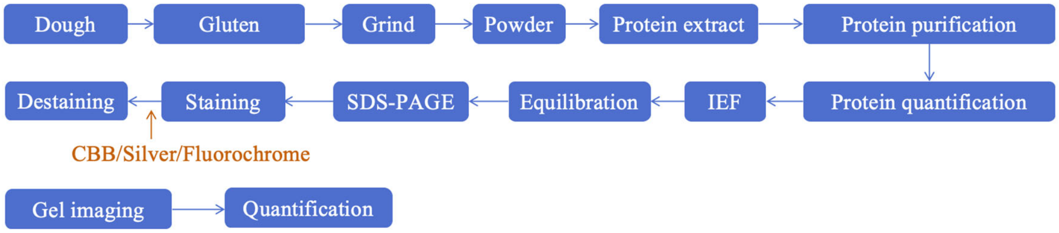

Fig. 1. Workflow of 2-DE for analysis of monomeric/subunit composition of gluten fractions.

References

Vensel, W. H., Tanaka, C. K., & Altenbach, S. B. (2014). Protein composition of wheat gluten polymer fractions determined by quantitative two-dimensional gel electrophoresis and tandem mass spectrometry. *Proteome Science*, *12*(1), 8. https://doi.org/10.1186/1477-5956-12-8

Wang, X., Appels, R., Zhang, X., Bekes, F., Torok, K., Tomoskozi, S., Diepeveen, D., Ma, W., & Islam, S. (2017). Protein-transitions in and out of the dough matrix in wheat flour mixing. *Food Chemistry*, 217, 542–551. https://doi.org/10.1016/j.foodchem.2016.08.060
