# Supplementary material for: Key Methodologies in Characterizing the Multi-Scale Structures of Gluten Proteins in Dough: A Comparative Review
Source: Biomolecules. 2026 Mar 3;16(3):382. doi: 10.3390/biom16030382 (PMC13023611; doi:10.3390/biom16030382)
Supplement: Supplementary file 1 [file biomolecules-16-00382-s001.zip › Supplementary File S4.pdf]

Supplementary material S4：

Analysis of monomeric/subunit composition of gluten fractions—size-exclusion high performance liquid chromatography

Principle

Size-exclusion high performance liquid chromatography (SE-HPLC) separates protein targets based on their molecular weight or, more specifically, hydrodynamic volume. As a result, a size-based elution curve is concluded, in which protein components are arranged in order of molecular weight against the elution time. The larger the size of a component, the earlier the location it occupied. Based on the retention time and area of an elution peak, the protein component could be identified, and its molecular weight and abundance could be estimated.

Apparatus

1. High-performance liquid chromatography system; equipped with UV detector set at 214 nm.

2. Size-exclusion chromatography column (TSKgel G4000SWXL, 7.8 mm×300 mm); used for molecular exclusion chromatography of gluten proteins.

3. Chromatography software (Open LAB, version 2.0); used for chromatographic peak integration and area calculation.

Reagents

1. Sodium phosphate buffer (PBS, 0.05 M, pH 6.8; prepared from NaH_2PO_4 and Na_2HPO_4): used for protein extraction and purification.

2. Sodium dodecyl sulfate (SDS): used at 1% (w/v) in the extraction buffer and 0.2% (w/v) in the elution buffer.

3. Dithiothreitol (DTT): used at 1% (w/v) in PBS buffer for complete reduction of gluten proteins.

Procedure

1. Dough is prepared by mixing 500 g of wheat flour (Nisshin Seifun, crude protein 8.5%, ash 0.34%) with 160 g of deionized water, followed by kneading using a mixer for 20 min at 139 rpm to produce a wheat dough. Fresh dough is washed with a 2% NaCl solution until the liquor becomes clear. This process is continued until the water used to rinse the dough no longer turns blue when tested with iodine solution, yielding wet gluten. The isolated wet gluten is freeze-dried, then ground and passed through an 80-mesh sieve to obtain gluten protein powder.

2. Weigh 15 mg of freeze-dried sample (gluten protein powder) and extract it at room temperature for 2 hours using 1 mL of sodium phosphate buffer (PBS, 0.05 M, pH 6.8) containing 1% SDS.

4. After centrifugation (5,000×g, 4 °C, 5 min), filter the collected supernatant through a 0.45 μm microporous filter.

5. Load 20 μL of the filtered supernatant onto a TSKG4000-SWXL column. The eluent is PBS (0.05 M, pH 6.8) containing 0.2% SDS, with a flow rate of 0.7 mL/min, column temperature at 30°C, and UV detection wavelength set at 214 nm. Record the elution curve using the Chromaster system to obtain the unreduced molecular exclusion chromatogram.

6. For complete reduction of samples, extract and analyze under the same conditions as the unreduced samples, except that PBS contains 1% DTT, to obtain the reduced molecular exclusion chromatogram.

7. Calculate the chromatographic peak area using Open LAB software.

6. Workflow diagram

An overview of the SE-HPLC workflow is shown in Fig. 1.

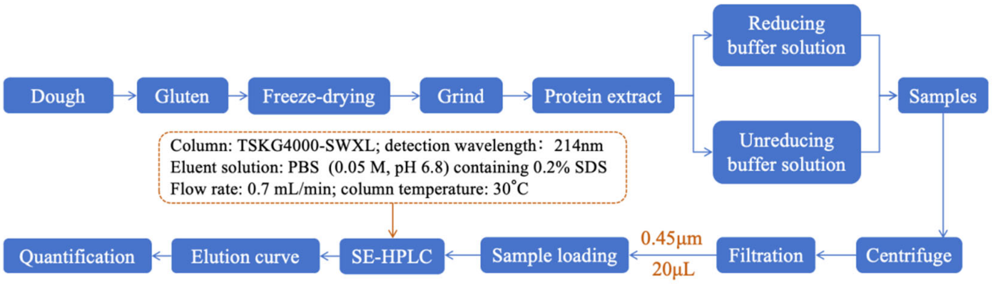

Fig. 1. Workflow of SE-HPLC for analysis of monomeric/subunit composition of gluten fractions.

References

Iwaki, S., Hayakawa, K., Fu, B.-X., & Otobe, C. (2021). Changes in hydrophobic interactions among gluten proteins during dough formation. *Processes*, *9*(7), 1244. https://doi.org/10.3390/pr9071244

Morel, M.-H., Pincemaille, J., Chauveau, E., Louhichi, A., Violleau, F., Menut, P., Ramos, L., & Banc, A. (2020). Insight into gluten structure in a mild chaotropic solvent by asymmetrical flow field-flow fractionation (AsFlFFF) and evidence of non-covalent assemblies between glutenin and ω-gliadin. *Food Hydrocolloids*, *103*, 105676. https://doi.org/10.1016/j.foodhyd.2020.105676
