# Supplementary material for: Key Methodologies in Characterizing the Multi-Scale Structures of Gluten Proteins in Dough: A Comparative Review
Source: Biomolecules. 2026 Mar 3;16(3):382. doi: 10.3390/biom16030382 (PMC13023611; doi:10.3390/biom16030382)
Supplement: Supplementary file 1 [file biomolecules-16-00382-s001.zip › Supplementary File S5.pdf]

Supplementary material S5：

Analysis of monomeric/subunit composition of gluten fractions—reversed-phase high-performance liquid chromatography

Principle

Reversed-phase high-performance liquid chromatography (RP-HPLC) separates proteins based on their differences in hydrophobicity, in which the elution orders of protein components are determined by the intensities of their hydrophobic interactions with the non-polar stationary phase of the applied C8 column. With a specific column, the higher the hydrophobicity of a protein component, the later it elutes from the column. In such cases, a hydrophobicity-based elution curve was concluded for all protein components against the elution time. The protein corresponding to each elution peak could be identified with retention time, and its abundance can be estimated with peak area.

Apparatus

1. High-performance liquid chromatography system (1260 Infinity II HPLC): used for RP-HPLC analysis with UV detection at 214 nm.

2. Chromatography column (Nucleosil 300-5 C8, 4.6×250 mm): used for gliadin and glutenin separation.

3. Chromatography software (OpenLAB, version 2.0): used for chromatographic peak integration and area calculation.

Reagents

1. Ethanol solution (60%, v/v): used for gliadin extraction.

2. Extraction buffer: 50% isopropanol aqueous solution, containing 0.05 mol/L Tris-HCl, 2 mol/L urea, and 1% dithiothreitol, pH 7.5; used for glutenin extraction.

3. Mobile phase: Phase A: Water containing 0.1% trifluoroacetic acid (v/v); Phase

B: Acetonitrile containing 0.1% trifluoroacetic acid (v/v).

Procedure

1. Preparation of samples

Dough is prepared by mixing 500 g of wheat flour (Nisshin Seifun, crude protein 8.5%, ash 0.34%) with 160 g of deionized water, followed by kneading using a mixer for 20 min at 139 rpm to produce a wheat dough. Fresh dough is washed with a 2% NaCl solution until the liquor becomes clear. This process is continued until the water used to rinse the dough no longer turns blue when tested with iodine solution, yielding wet gluten. The isolated wet gluten is freeze-dried, then ground and passed through an 80-mesh sieve to obtain gluten protein powder.

Accurately weigh 100 mg of gluten protein into a 10 mL centrifuge tube and add 5 mL of 60% ethanol aqueous solution. Mix well, then perform extraction in a water bath (20 °C, 20 minutes), and centrifuge (10,000×g, 20 °C, 10 min). Repeat the extraction twice and combine the supernatants. This will be the gliadin protein.

Under N_2 conditions, add 3 mL of the extraction buffer (50% isopropanol aqueous solution, containing 0.05 mol/L Tris-HCl, 2 mol/L urea, and 1% DTT, pH 7.5) to the remaining precipitate. Stir (20 °C, 1 h) and then centrifuge (10,000×g, 20 °C, 10 min). After extracting once more, add 4 mL of the extraction buffer and extract once more. Combine the 3 supernatants to obtain glutenin protein.

2. RP-HPLC Analysis

Sample pretreatment: the supernatants of the gliadin protein and glutenin protein are filtered through a 0.22 μm organic filter membrane for HPLC analysis.

Chromatographic column: Nucleosil 300-5 C8 chromatographic column (4.6 × 240 mm).

Mobile phase: Phase A: Water containing 0.1% trifluoroacetic acid (v/v); Phase B: Acetonitrile containing 0.1% trifluoroacetic acid (v/v).

Elution program: Gradient elution for 50 minutes, with the concentration of B phase increasing linearly from 24% to 56%.

Flow rate: 1 mL/min; injection volume: 20 μL; column temperature: 50 ℃; detection wavelength: 214 nm.

Calculate the chromatographic peak area using Open LAB software. The content of each subunit is calculated as the proportion of its peak area to the total peak area.

3. Workflow diagram

An overview of the RP-HPLC workflow is shown in Fig. 1.

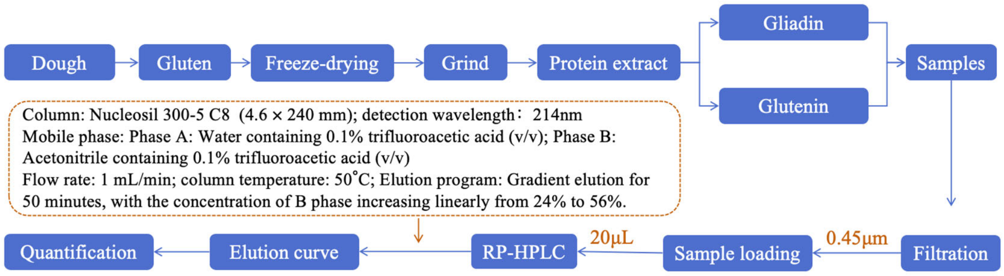

Fig. 1. Workflow of RP-HPLC for analysis of monomeric/subunit composition of gluten fractions.

References

Kieffer, R., Schurer, F., Köhler, P., & Wieser, H. (2007). Effect of hydrostatic pressure and temperature on the chemical and functional properties of wheat gluten: Studies on gluten, gliadin, and glutenin. *Journal of Cereal Science*, *45*, 285-292. https://doi.org/10.1016/j.jcs.2006.09.008

Naeem, H. A., & Sapirstein, H. D. (2007). Ultra-fast separation of wheat glutenin subunits by reversed-phase HPLC using a superficially porous silica-based column. *Journal of Cereal Science*, *46*, 157-168. https://doi.org/10.1016/j.jcs.2007.01.002
