# Supplementary material for: Key Methodologies in Characterizing the Multi-Scale Structures of Gluten Proteins in Dough: A Comparative Review
Source: Biomolecules. 2026 Mar 3;16(3):382. doi: 10.3390/biom16030382 (PMC13023611; doi:10.3390/biom16030382)
Supplement: Supplementary file 1 [file biomolecules-16-00382-s001.zip › Supplementary File S6.pdf]

Supplementary material S6：

Analysis of monomeric/subunit composition of gluten fractions—Proteomics

Principle

Roughly, the proteomic workflow chiefly consists of the following procedures: (1) sample preparation, (2) protein extraction, separation, and digestion, (3) peptide separation and mass analysis, and (4) data analysis.

Apparatus

1. 2-DE electrophoresis system (Bio-Rad, model: PROTEAN II xi Cell); used for protein separation before in-gel digestion.

2. Discovery DSC18 SPE columns: used for peptide purification.

3. ZipTip C18 columns: used for microscale peptide desalting.

4. Nano-LC system: ReproSil-Pur C18-AQ columns (3 µm for analytical, 5 µm for trap); analytical column 75 µm×15 cm; trap column 150 µm×2 cm; used for nanoflow RP-HPLC separation of peptides.

5. High-resolution mass spectrometer; used for MS (60,000@ m/z 400) and DDA MS/MS acquisition.

6. Data analysis software: OpenLAB for LC; Thermo, Proteome Discoverer, optional for MS.

Reagents

1. Tris-HCl buffer; used for enzymatic digestion.

2. Urea solution (0.04 mol/L in digestion mix); used for protein denaturation during solution digestion.

3. Chymotrypsin: prepared at 0.02 mg/mL; used for conventional enzymatic hydrolysis at 37 °C for 24 h.

4. Trypsin: diluted to 20 ng/μL in 25 mM NH_4HCO_3.

5. Trifluoroacetic acid (TFA, 0.1%, v/v): used to stop digestion and in SPE/ZipTip solvents.

6. Silver-stain decolorization components: Potassium ferricyanide ($K_3[Fe(CN)_6]$) and Sodium sulfite (Na_2SO_3) are freshly mixed 30 mM: 100 mM (1:1).

7. Acetonitrile (ACN, 60/70%, v/v): used for gel plug dehydration and peptide elution.

8. Solid-phase extraction reagent: equilibrated with 0.1% TFA, peptides eluted with 60% or 70% ACN in 0.1% TFA

Procedure

1. Preparation of samples

Dough is prepared by mixing 500 g of wheat flour (Nisshin Seifun, crude protein 8.5%, ash 0.34%) with 160 g of deionized water, followed by kneading using a mixer for 20 min at 139 rpm to produce a wheat dough. The dough is freeze-dried and then ground through a 100-mesh sieve.

2. Protein extraction, separation, and digestion

2.1 Protein extraction

Albumin/globulin (ALGL), gliadin, and glutenin fractions are obtained according to Osborne classification (Supplementary Material 1).

2.2 Protein separation

Before digestion, the protein components can be separated by two-dimensional gel electrophoresis (2-DE) and silver staining according to Supplementary Material 3.

2.3 Protein digestion

2.3.1 Conventional enzymatic hydrolysis

Take 1 mg protein, add 0.02 mg/mL chymotrypsin dissolved in 0.04 mol/L urea,

0.1 mol/L Tris-HCl (pH 7.8) solution. The samples are incubated at 37 °C for 24 h for enzymatic hydrolysis, and then 3 μL TFA is added to terminate the reaction.

2.3.2 Enzymatic digestion of protein spots on 2-DE gel

Gel cutting: the differential protein spots on the 2-DE gel are dug out and placed into a 1.5 mL EP tube.

Water washing: add ultrapure water to wash for 1 min, remove the supernatant by centrifugation (10,000×*g*, 30 min, 4℃), and repeat water washing twice.

Destained: silver-stained destained solution is added until the protein spots are completely decolorized, and the destained solution is removed by suction with a pipette gun.

Water washing: add ultrapure water and wash 3 times to remove residual decolorization liquid.

Dehydration: Add 100% acetonitrile (ACN) until the colloidal particles turn white to complete the dehydration.

Digestion: the trypsin is diluted to 20 ng/μL with 25 mM NH_4HCO_3; An appropriate amount of trypsin and chymotrypsin solution is added to each tube, and an ice bath is performed for 30 minutes and incubated at 37℃ overnight.

3. Purification and separation of peptide segments

3.1 Peptide purification

3.1.1 Solid phase extraction column purification

Column: commonly used Discovery DSC-18 SPE solid-phase extraction column for peptide purification.

Column activation: add 1.0 mL of methanol, discard the waste solution, and repeat twice.

Equilibration: add 1.0 mL 0.1% TFA (v/v), discard the waste solution, and repeat

twice.

Sample loading: 1.0 mL of enzymatic hydrolysate is added to the column and left for 5 min to fully adsorb the peptide.

Leaching: successively use 1.0 mL 0.1% TFA (v/v) and 1.0 mL 5% ACN/0.1% TFA (v/v) to leach the column and discard the waste liquid.

Elution: the peptide is eluted with 0.4 mL 60% ACN/0.1% TFA (v/v), the eluate is collected in a new tube and concentrated under vacuum until dry.

3.1.2 Purification by ZipTip method

Column activation: suck 10 μL 100% ACN, pass through the ZipTip column, discard the waste liquid, and repeat for 2 times. Post acidification: suck 10 μL of 0.1% trifluoroacetic acid (TFA), pass through the post, discard the waste liquid, and repeat for 2 times.

Adsorption sample: the peptide sample is blown and aspirated 15 times with the ZipTip column to make the peptide adsorbed on the column.

Remove impurities: absorb 10 μL of 0.1% TFA, pass through the column, discard the waste liquid, and repeat for 2 times.

Elution sample: 10 μL of eluate containing 0.1% TFA and 70% ACN is aspirated, passed through the column, and the eluate is collected into the new EP tube.

Vacuum drying: the eluted peptide solution is vacuum-drained until LC-MS /MS analysis.

3.2 Peptide separation

Part of the enzymatic peptide is used for high-performance liquid chromatography-mass spectrometry analysis. A C18 reverse-phase column with an inner diameter of 75 μm and a length of 15 cm and a packing of 3 μm ReproSil-Pur C18-AQ is used as the chromatographic analysis column, and a loading column with an inner diameter of 150 μm, a length of 2 cm, and a packing of 5 μm ReproSil-Pur

C18-AQ is used.

4. Mass analysis

Chromatographic analysis is performed using a 150-min gradient and a flow rate of 300 nL/min. The Q-Exactive MS transmission tube temperature is 220ºC, the spray voltage is 2.0 kV, the full scan resolution is 60000@m/z400, the scan range is 300-1800, and the 10 highest intensity ions are selected for secondary fragmentation in each full scan. The trigger intensity of secondary mass spectrometry is 500, the separation window is 2 amu, the maximum ion injection time of primary mass spectrometry and secondary mass spectrometry is 500 ms and 150ms, respectively, and the continuous repetition time and exclusion duration are 30 ms and 120 s, respectively.

5. Data analysis

Gluten protein annotation and enrichment analysis are performed using the UniProt database (http://www.uniprot.org/). GO analysis of proteins is performed according to molecular function. KEGG (Kyoto Encyclopedia of Genes and Genomes) (http://www.kegg.jp/), COG (Clusters of Orthologous Groups of proteins) (http://www.ncbi.nlm.nih.gov/COG) analysis. MeV (4.8.0) software is used to perform hierarchical cluster analysis of differential proteins

6. Workflow diagram

An overview of the Proteomics workflow used to analyze of monomeric/subunit composition of gluten fractions is shown in Fig. 1.

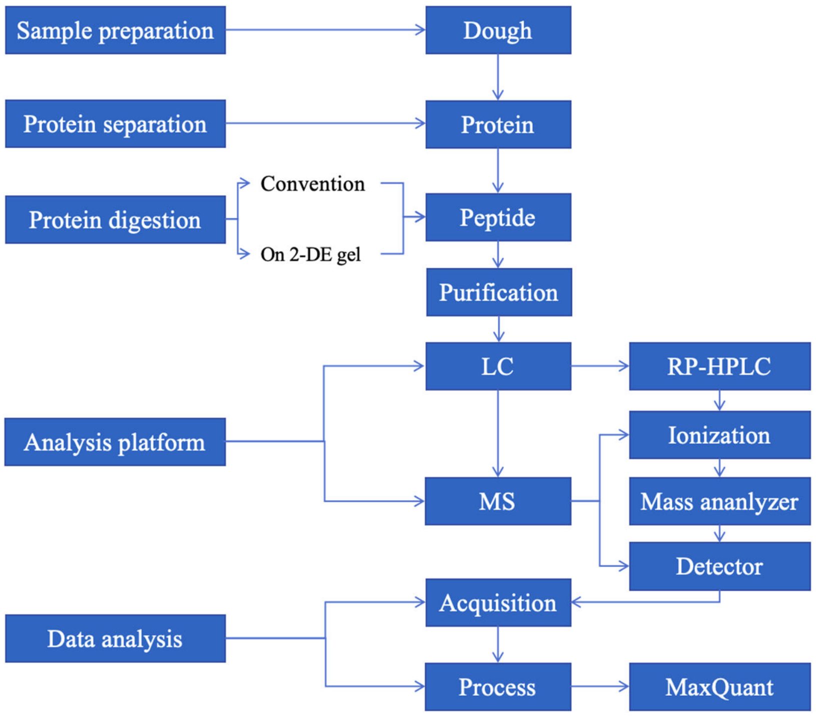

Fig. 1. Proteomics workflow used to analyze monomeric/subunit composition of gluten fractions.

References

Afzal, M., Pfannstiel, J., Zimmermann, J., Bischoff, S. C., Würschum, T., & Longin, C. F. H. (2020). High-resolution proteomics reveals differences in the proteome of spelt and bread wheat flour, representing targets for research on wheat sensitivities. *Scientific Reports*, *10*, 14677. https://doi.org/10.1038/s41598-020-71712-5

Dupont, F. M., Vensel, W. H., Tanaka, C. K., Hurkman, W. J., & Altenbach, S. B. (2011). Deciphering the complexities of the wheat flour proteome using quantitative two-dimensional electrophoresis, three proteases and tandem mass spectrometry. *Proteome Science*, *9*, 10. https://doi.org/10.1186/1477-5956-9-10

Schirmer, T. M., Ludwig, C., & Scherf, K. A. (2023). Proteomic characterization of wheat protein fractions taken at different baking conditions. *Journal of Agricultural and Food Chemistry*, *71*, 12899-12909. https://doi.org/10.1021/acs.jafc.3c02100
