# Supplementary material for: Key Methodologies in Characterizing the Multi-Scale Structures of Gluten Proteins in Dough: A Comparative Review
Source: Biomolecules. 2026 Mar 3;16(3):382. doi: 10.3390/biom16030382 (PMC13023611; doi:10.3390/biom16030382)
Supplement: Supplementary file 1 [file biomolecules-16-00382-s001.zip › Supplementary File S7.pdf]

Supplementary material S7:

Structure analysis of gluten proteins—Fourier transform infrared spectroscopy

Principle

Fourier transform infrared spectroscopy (FT-IR) determines protein secondary structures by detecting bond vibrations, with amide I (1700-1600 cm^{-1}) and amide III (1330-1220 cm^{-1}) being the most informative regions. The workflow involves spectrum acquisition, processing, band assignment, and quantitative calculation. Spectrum processing typically includes water subtraction, baseline correction, smoothing, normalization, second-derivative analysis, and Fourier self-deconvolution. The proportion of each secondary structural element (α-helices, β-sheets, β-turns, and random coils) is finally obtained by normalizing the area of its assigned band to the total area of all resolved components.

Apparatus

1. FT-IR spectrometer (Nicolet iS50, equipped with MCT/A detector and diamond ATR accessory): used for acquisition of infrared spectra of gluten proteins in the range of 400-4000 cm^{-1}.

2. Nitrogen generator: used to purge spectrometer chamber to prevent water vapor interference in the amide I and III regions.

3. OMNIC software: used for data collection, baseline correction, smoothing, normalization, and deconvolution.

4. PeakFit software: used for curve fitting of FT-IR spectra to calculate the relative proportion of secondary structural elements.

Procedure

1. Preparation of Samples

Dough is prepared by mixing 500 g of wheat flour (Nisshin Seifun, crude protein 8.5%, ash 0.34%) with 160 g of deionized water, followed by kneading using a mixer

(Hobart, N50) for 20 min at 139 rpm to produce a wheat dough. The dough is freeze-dried and then ground through a 100-mesh sieve.

2. Instrument selection

Select ATR-FTIR equipped with mercury-cadmium telluride (MCT/ a) detector and ATR accessory.

3. Spectrum acquisition

The spectrometer is continuously cleaned with nitrogen to avoid interference from water vapor absorption in the amide region of the protein.

Sample (0.5g) of lyophilized dough powder is transferred to the crystal of the attenuated total reflection attachment, and the screw is rotated and tightened to achieve a table-pressing effect.

Spectra are collected at a resolution of 4 cm^{-1} in the frequency range from 400 to 4000 cm^{-1}.

4. Spectrum process

The interferograms of 256 scans are encoded and Fourier transformed using the Happ/Ganzel apodization function.

The empty crystal is used to detract the background signal, and the infrared spectrum is obtained by recording the data using OMNIC software, which is processed by baseline correction, Gaussian smoothing and normalization in turn.

The baseline correction can avoid the ring oscillation phenomenon on both sides of the spectrum obtained by deconvolution. If the ring oscillation phenomenon still occurs, the resolution enhancement factor is selected too large. At this time, the resolution enhancement factor should be appropriately reduced to obtain the correct results.

Smoothing can attenuate the effect of noise on deconvolution.

Normalization can eliminate sample size differences, reduce baseline drift and

background interference, improve data comparability and accuracy of analysis.

5. Quantitative calculation

Fourier self-deconvolution and the second derivative technique are used to determine the number and location of overlapping bands. For Fourier self-deconvolution, the choice of two important parameters, bandwidth and enhancement factor, is based on the relationship between the second derivative and the original spectrum, and the parameters are considered appropriate when the number of peaks and their positions in both curves matched.

Finally, PeakFit software is used for Gaussian/Lorentzian curve fitting (Fig. 2). The curve-fitting procedure is as follows: (1) the frequency of the peaks is manually adjusted by moving the cursor to the desired wave number determined by the self-deconvolution and/or second-derivative resolution enhancement techniques described above, (2) the peak is adjusted iteratively to achieve the best fit, and (3) the area under the peak is calculated for individual bands corresponding to specific secondary structure motifs. (4) The percentage of specific secondary structure is calculated by dividing the designated band area by the total area.

6. Workflow diagram

An overview of the FT-IR workflow is shown in Fig. 1.

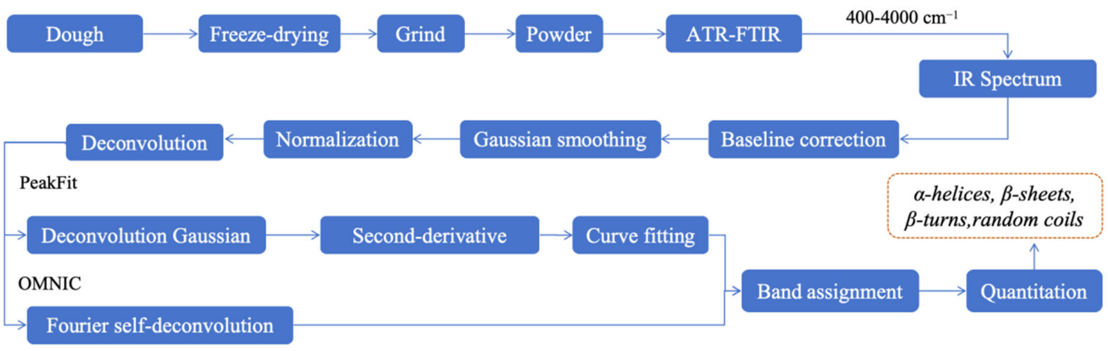

Fig. 1. Workflow of FT-IR for structure analysis of gluten proteins.

References

Cai, S., & Singh, B. R. (2004). A distinct utility of the amide III infrared band for secondary structure estimation of

aqueous protein solutions using partial least squares methods. *Biochemistry*, *43*(9), 2541–2549. https://doi.org/10.1021/bi030149y

Fevzioglu, M., Ozturk, O. K., Hamaker, B. R., & Campanella, O. H. (2020). Quantitative approach to study secondary structure of proteins by FT-IR spectroscopy, using a model wheat gluten system. *International Journal of Biological Macromolecules*, *164,* 2753–2760. https://doi.org/10.1016/j.ijbiomac.2020.07.299

Kłosok, K., Welc-Stanowska, R., & Nawrocka, A. (2023). Changes in the conformation and biochemical properties of gluten network after phenolic acid supplementation. *Journal of Cereal Science*, *110*, 103651. https://doi.org/10.1016/j.jcs.2023.103651

Wang, P., Zou, M., Gu, Z., & Yang, R. (2018). Heat-induced polymerization behavior variation of frozen-stored gluten. *Food Chemistry*, *255*, 242–251. https://doi.org/10.1016/j.foodchem.2018.02.047
