# Supplementary material for: Key Methodologies in Characterizing the Multi-Scale Structures of Gluten Proteins in Dough: A Comparative Review
Source: Biomolecules. 2026 Mar 3;16(3):382. doi: 10.3390/biom16030382 (PMC13023611; doi:10.3390/biom16030382)
Supplement: Supplementary file 1 [file biomolecules-16-00382-s001.zip › Supplementary File S8.pdf]

Supplementary material S8：

Structure analysis of gluten proteins—Fourier transform Raman spectroscopy

Principle

Fourier transform Raman spectroscopy (FT-Raman) characterizes protein structures by detecting vibrational modes of chemical bonds. The amide I (1700-1600 cm^{-1}) and amide III (1220-1330 cm^{-1}) bands provide information on secondary structure elements such as α-helix, β-sheet, β-turn, and random coil. In terms of tertiary structure, FT-Raman detects disulfide bond conformations through SS stretching bands (500-550 cm^{-1}) and evaluates the microenvironment of aromatic residues. The tryptophan band (~755 cm^{-1}) and the tyrosine doublet (~830/850 cm^{-1}) indicate local polarity. Thus, FT-Raman enables non-destructive assessment of both secondary structure composition and tertiary structural features of gluten proteins.

Apparatus

1. Fourier transform infrared spectrometer with FT-Raman module: equipped with an InGaAs detector and a CaF_2 beam splitter, used for Raman spectral acquisition of hydrated gluten and gluten protein powder.

2. OMNIC software: used for data collection, baseline correction, smoothing, normalization, and deconvolution.

3. PeakFit software; used for curve fitting of FT-Raman spectra to calculate the relative proportion of secondary structural elements and disulfide bond conformation.

Procedure

1. Preparation of Samples

Dried powder of dough: Dough is prepared by mixing 500 g of wheat flour (Nisshin Seifun, crude protein 8.5%, ash 0.34%) with 160 g of deionized water, followed by kneading using a mixer (Hobart, N50) for 20 min at 139 rpm to produce a wheat dough. The dough is freeze-dried and then ground through a 100-mesh sieve.

Hydrated gluten: take fresh dough and wash it repeatedly with 2% (w/v) NaCl solution until the filtrate is clear. The hydrated gluten sample is placed in 2% NaCl solution (which inhibits the enzymatic hydrolysis of gluten) and directly used for FT-Raman measurement in solid form.

Notes: Water has a very weak Raman signal, so it is possible to directly measure the hydrated dough gluten protein.

Gluten protein powder: the hydrated gluten protein is freeze-dried and then ground through a 100-mesh sieve.

2. Instrument selection

FT-Raman spectra are obtained on the FT-Raman module of a Nicolet 6700 Fourier transform infrared spectrometer with a commonly used InGaAs detector and CaF2 beam splitter.

3. Spectrum acquisition

The prepared sample is placed in a stainless-steel cube and illuminated with a 1064nm Nd: YAG excitation laser. The maximum laser power is 1W. Spectra are recorded in the range of 3500 to 150 cm^{-1}, with an average of 256 scans per spectrum at 8cm^{-1} resolution. The analyzed spectra are averaged over the five registered spectra.

4. Spectrum process, band assignment, and quantitative calculation

Spectral data from sample scans are baseline corrected and normalized for the 1003 cm^{-1} phenylalanine band using OMNIC software.

When analyzing the data, the wave intensity changes or the second derivative band area of the structure at all levels can be selected to analyze according to the research content.

We recorded the characteristic band intensities at 755, 830, and 854 cm^{-1}, and used the ratio of I_{854}/I_{830} and the band density of I_{755} to characterize the microenvironment of tyrosine and tryptophan.

Regarding the disulfide bond bridge region (490-550 cm^{-1}) and secondary structure elements (1700-1600 cm^{-1}, 1220-1330 cm^{-1}), the empty crystal is used to detract the

background signal, and the infrared spectrum is obtained by recording the data using OMNIC software, which is processed by baseline correction, Gaussian smoothing and normalization in turn. Fourier deconvolution and the second derivative technique are used to determine the number and location of overlapping bands.

5. Quantitative calculation

Finally, regarding the disulfide bond bridge region (490-550 cm^{-1}) and secondary structure elements (1700-1600 cm^{-1}, 1220-1330 cm^{-1}), PeakFit v4.12 software is used for Gaussian/Lorentzian curve fitting. The curve-fitting procedure is as follows: (1) the frequency of the peaks is manually adjusted by moving the cursor to the desired wave number determined by the deconvolution and/or second-derivative resolution enhancement techniques described above, (2) the peak is adjusted iteratively to achieve the best fit, and (3) the area under the peak is calculated for individual bands corresponding to specific secondary structure motifs. (4) The percentage of specific secondary structure is calculated by dividing the designated band area by the total area.

6. Workflow diagram

An overview of the FT-Raman workflow is shown in Fig. 1.

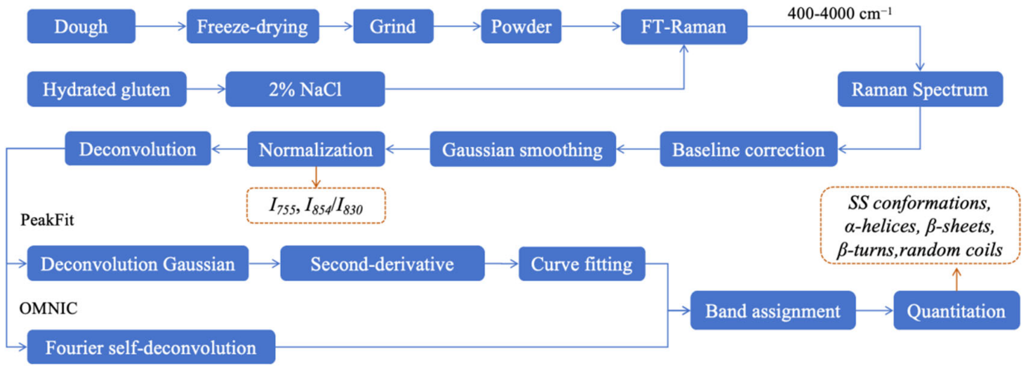

Fig. 1. Workflow of Fourier transform Raman spectroscopy (FT-Raman) for structure analysis of gluten proteins.

References

Herrero, A. M., Carmona, P., Cofrades, S., & Jiménez-Colmenero, F. (2008). Raman spectroscopic determination of structural changes in meat batters upon soy protein addition and heat treatment. *Food Research International*, 41, 765-772. https://doi.org/10.1016/j.foodres.2008.06.001

Lin, Q., Shen, H., Ma, S., Zhang, Q., Yu, X., & Jiang, H. (2023). Morphological Distribution and Structure Transition of Gluten Induced by Various Drying Technologies and Its Effects on Chinese Dried Noodle Quality Characteristics. *Food and Bioprocess Technology*, *16*(6), 1374–1387. https://doi.org/10.1007/s11947-023-02993-7

Nawrocka, A., Szymańska-Chargot, M., Miś, A., Ptaszyńska, A. A., Kowalski, R., Waśko, P., & Gruszecki, W. I. (2015). Influence of dietary fibre on gluten proteins structure-a study on model flour with application of FT-Raman spectroscopy. *Journal of Raman Spectroscopy*, 46, 309-316. https://doi.org/10.1002/jrs.4648

Sadat, A., Corradini, M. G., & Joye, I. J. (2019). Molecular spectroscopy to assess protein structures within cereal systems. *Current Opinion in Food Science*, *25*, 42-51. https://doi.org/10.1016/j.cofs.2019.02.001

Zhou, Y., Zhao, D., Foster, T. J., Liu, Y., Wang, Y., Nirasawa, S., Tatsumi, E., & Cheng, Y. (2014). Konjac glucomannan-induced changes in thiol/disulphide exchange and gluten conformation upon dough mixing. Food Chemistry, *143*, 163-169. https://doi.org/10.1016/j.foodchem.2013.07.088
