# Supplementary material for: Key Methodologies in Characterizing the Multi-Scale Structures of Gluten Proteins in Dough: A Comparative Review
Source: Biomolecules. 2026 Mar 3;16(3):382. doi: 10.3390/biom16030382 (PMC13023611; doi:10.3390/biom16030382)
Supplement: Supplementary file 1 [file biomolecules-16-00382-s001.zip › Supplementary File S9.pdf]

Supplementary material S9:

Analysis of the covalent bonds—spectrophotometric method

Principle

The amount of SH ($F_{\text{-SH}}$) is typically determined directly with samples using Ellman's reagent by a spectrophotometric method at 412 nm. However, to determine the amount of SS, the sample is first subjected to a reduction to completely convert SS into SH, and then total SH ($T_{\text{-SH}}$) is determined. The amount of SS is derived as the half value of the residue by subtracting $F_{\text{-SH}}$ from $T_{\text{-SH}}$. To reduce SS, β-mercaptoethanol (β-ME) and tris(2-carboxyethyl)phosphine (TCEP) methods are often applied.

Apparatus

1. UV-Vis spectrophotometer: set to 412 nm for sulfhydryl quantification.

2. Centrifuge column (paper-based filter membrane, pore size about 10 μm, Pierce products): loaded with TCEP reducing gel.

Reagents

1. Tris-Gly buffer: containing 0.09 M Tris-HCl, 0.09 M glycine, and 0.04 M ethylenediaminetetraacetic acid disodium dihydrate, pH=8.0.

2. The reaction buffer: prepared by dissolving 5 M guanidine hydrochloride (GuHCl) in the Tris-Gly buffer.

3. Ellman's reagent: by dissolving 40 mg of 5,5'-dithiobis (2-nitrobenzoic acid) (DTNB) in 10 mL of Tris-Gly buffer.

4. β-ME solution: containing 4 mL urea, 5 M GuHCl solution, and 50 μL β-ME.

Procedure

1. Preparation of Samples

Dough is prepared by mixing 500 g of wheat flour (Nisshin Seifun, crude protein 8.5%, ash 0.34%) with 160 g of deionized water, followed by kneading using a mixer for 20 min at 139 rpm to produce a wheat dough. The dough is freeze-dried and then

ground through a 100-mesh sieve.

Weigh 100 mg freeze-dried dough powder, add 1.0 mL of the reaction buffer, vortex and shake for 1 min, and incubate for 30 min at 25 °C in the dark on a shaker. Then, the mixture is centrifuged (1,3600×g, 4 °C, 25 min).

After 50 μL of the supernatant is diluted with 500 mL the reaction buffer, 10 mL of Ellman's reagent is added. After vortex shaking, the samples are incubated for 20 min at 25 °C in the dark. Absorbance is measured at 412 nm using a UV-visible spectrophotometer with a reagent blank as a control.

2. *β*-ME reduction method

For the determination of total sulfhydryl content, 1 mL of the supernatant obtained by the above method is mixed with *β*-ME solution.

The mixture is incubated at 25 °C for 1 hour. Subsequently, 5 mL trichloroacetic acid (13%, w/v) is added, and the incubation is continued for 1 hour at 25 °C. After centrifugation (10,000 rpm, 25 °C, 10 min), the precipitate is re-suspended, dissolved, and mixed with Ellman's reagent.

3. TCEP reduction method

The total sulfhydryl content is determined by the tris (2-carboxyethyl) phosphine (TCEP) reduction gel method. A centrifuge column (paper-based filter membrane, pore size about 10 μm, Pierce products) loaded with TCEP reducing gel is equilibrated with the reaction buffer in a microcentrifuge tube.

Subsequently, 70 μL of the supernatant obtained by the above method is added to the centrifugal column, vortexed and shaken for 30 seconds, and incubated on a shaker for 20 min at room temperature. The samples are centrifuged at 2,447×*g* for 2 min, and the effluent is collected as the reduced sample.

4. Workflow diagram

An overview of the analysis of the content of the SH and SS workflow is shown in Fig. 1.

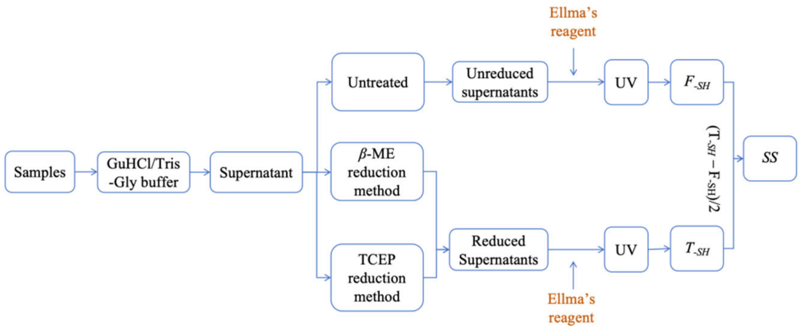

Fig. 1. Workflow for analyzing the content of SH and SS.

Result presentation

The free sulfhydryl, total sulfhydryl, and disulfide bond content are calculated by measuring the absorbance at 412 nm using equations (1), (2), and (3), respectively.

$$F_{\text{-SH}}\ (\text{mmol/g}) = 73.53 \times A_{412} \times D/C \qquad (1)$$

$$T_{\text{-SH}}\ (\text{mmol/g}) = 73.53 \times A_{412} \times D/C \qquad (2)$$

$$SS\ (\text{mmol/g}) = (T_{\text{-SH}} - T_{\text{-SH}})/2 \qquad (3)$$

In equations (1) and (2), A_{412} represents the absorbance at 412 nm, D stands for the dilution factor, and C denotes the sample concentration (mg/mL).

References

Gao, J., Koh, A. H. S., Tay, S. L., & Zhou, W. (2017). Dough and bread made from high- and low-protein flours by vacuum mixing: Part 1: Gluten network formation. *Journal of Cereal Science*, *74*, 288-295. https://doi.org/10.1016/j.jcs.2017.03.008

Lin, Q., Shen, H., Ma, S., Zhang, Q., Yu, X., & Jiang, H. (2023). Morphological distribution and structure transition of gluten induced by various drying technologies and its effects on Chinese dried noodle quality characteristics. *Food and Bioprocess Technology*, *16*, 1374-1387. https://doi.org/10.1007/s11947-023-02993-7

Liu, H., Liang, Y., Zhang, S., Liu, M., He, B., Wu, X., Yin, H., Zhang, X., & Wang, J. (2024). Physicochemical properties and conformational structures of pre-cooked wheat gluten during freeze-thaw cycles affected by curdlan. *Food Hydrocolloids*, *147*, 109381. https://doi.org/10.1016/j.foodhyd.2023.109381

Zhang, M. L., Ma, M., Jia, R. B., Yang, T. B., Sun, Q. J., & Li, M. (2022). Delineating the dynamic transformation of gluten morphological distribution, structure, and aggregation behavior in noodle dough induced by mixing

and resting. *Food Chemistry*, *386*, 132853. https://doi.org/10.1016/j.foodchem.2022.132853
