# Supplementary material for: Key Methodologies in Characterizing the Multi-Scale Structures of Gluten Proteins in Dough: A Comparative Review
Source: Biomolecules. 2026 Mar 3;16(3):382. doi: 10.3390/biom16030382 (PMC13023611; doi:10.3390/biom16030382)
Supplement: Supplementary file 1 [file biomolecules-16-00382-s001.zip › Supplementary File S10.pdf]

Supplementary material S10：

Structure analysis of gluten proteins—fluorescence spectroscopy

Principle

For fluorescence spectroscopy, it probes the tertiary and quaternary structures of proteins by mainly measuring the intrinsic fluorescence of tryptophan (Trp) residue, due to its dominant contribution to the total fluorescence of protein and high sensitivity to microenvironment polarity. Typically, Trp residues present the maximum emission peak around 350 nm. It is found that the position and fluorescence intensity of this peak highly correlate with the states of advanced structures of the protein. A shift toward lower wavelengths (blue shift) with an increase in peak intensity indicates a more compact structure, whereas a shift toward longer wavelengths (red shift) with a decrease in peak intensity reflects that the protein is undergoing loosening or unfolding.

Apparatus

1. Fluorescence spectrophotometer: used for fluorescence spectral acquisition of gluten protein.

2. Quartz cuvette; used for fluorescence measurement.

3. FL Solutions: used for blank subtraction and spectral correction.

4. OriginPro 2023: used for spectral analysis of emission maxima and fluorescence intensity.

Reagents

1. Phosphate buffer (0.01 mol/L, pH 7.0): used to extract the gluten protein sample.

Procedure

1. Preparation of Samples

Dried powder of dough: Dough is prepared by mixing 500 g of wheat flour (Nisshin Seifun, crude protein 8.5%, ash 0.34%) with 160 g of deionized water, followed by

kneading using a mixer for 20 min at 139 rpm to produce a wheat dough. Take fresh dough and wash it repeatedly with 2% (w/v) NaCl solution until the filtrate is clear. The hydrated gluten sample is freeze-dried and then ground through a 100-mesh sieve.

The freeze-dried gluten protein powder (50 mg) is dissolved in 25 mL of phosphate buffer. The solution is vortexed for 1 min to ensure complete dispersion, followed by centrifugation (8,000 rpm, 4 °C, 10 min) to remove insoluble fractions. The resulting supernatant was collected as the test sample.

2. Measurement procedure

Fluorescence spectra of gluten proteins were recorded using a fluorescence spectrophotometer equipped with a thermostated cell holder maintained at 25 °C. The excitation wavelength was set at 280 nm, and emission spectra were scanned from 250 to 480 nm with both excitation and emission slit widths fixed at 5 nm. The photomultiplier tube voltage was adjusted to 700 V, and the scan speed was 1200 nm/min. For measurements, 2-3 mL of the protein sample was loaded into a 1 cm quartz cuvette, and spectra were recorded under the above conditions. Each sample was analyzed in triplicate to ensure reproducibility.

3. Data processing

The raw fluorescence spectra were first corrected by subtracting the buffer blank using the instrument's software. The corrected data were subsequently analyzed with OriginPro 2023 to determine emission maxima (λ_{max}) shifts and fluorescence intensity changes.

4. Workflow diagram

An overview of the fluorescence spectroscopy workflow is shown in Fig. 1.

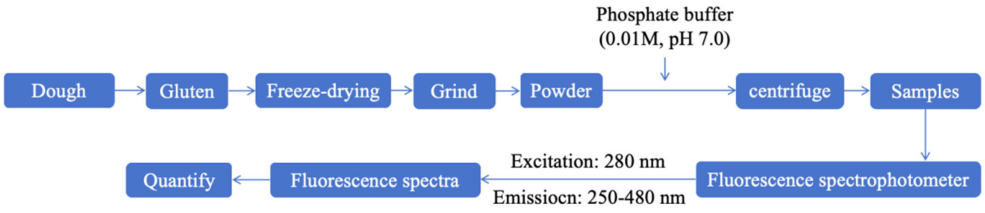

Fig. 1. Workflow of fluorescence spectroscopy for the Structure analysis of gluten proteins.

References

Rodrigues, F. H. d. S., Delgado, G. G., Costa, T. S. d., & Tasic, L. (2023). Applications of fluorescence spectroscopy in protein conformational changes and intermolecular contacts. *BBA Advances*, *3*, 100091. https://doi.org/10.1016/j.bbadva.2023.100091
